# Interleukin-36 Cytokines in Infectious and Non-Infectious Lung Diseases

**DOI:** 10.3389/fimmu.2021.754702

**Published:** 2021-11-23

**Authors:** Hernán F. Peñaloza, Rick van der Geest, Joel A. Ybe, Theodore J. Standiford, Janet S. Lee

**Affiliations:** ^1^ Acute Lung Injury Center of Excellence, Division of Pulmonary, Allergy, and Critical Care Medicine, Department of Medicine, University of Pittsburgh, Pittsburgh, PA, United States; ^2^ Department of Environmental and Occupational Health, School of Public Health, Indiana University, Bloomington, IN, United States; ^3^ Division of Pulmonary and Critical Care Medicine, University of Michigan, Ann Arbor, MI, United States; ^4^ Vascular Medicine Institute, University of Pittsburgh, Pittsburgh, PA, United States

**Keywords:** interleukin-36 cytokines, IL-1Rrp2, host/microbe proteases, inflammatory response amplification, lung infectious diseases, lung inflammation

## Abstract

The IL-36 family of cytokines were identified in the early 2000’s as a new subfamily of the IL-1 cytokine family, and since then, the role of IL-36 cytokines during various inflammatory processes has been characterized. While most of the research has focused on the role of these cytokines in autoimmune skin diseases such as psoriasis and dermatitis, recent studies have also shown the importance of IL-36 cytokines in the lung inflammatory response during infectious and non-infectious diseases. In this review, we discuss the biology of IL-36 cytokines in terms of how they are produced and activated, as well as their effects on myeloid and lymphoid cells during inflammation. We also discuss the role of these cytokines during lung infectious diseases caused by bacteria and influenza virus, as well as other inflammatory conditions in the lungs such as allergic asthma, lung fibrosis, chronic obstructive pulmonary disease, cystic fibrosis and cancer. Finally, we discuss the current therapeutic advances that target the IL-36 pathway and the possibility to extend these tools to treat lung inflammatory diseases.

## Historical Perspective of IL-36 Cytokines Discovery and Functional Studies

IL-36 cytokines are members of the IL-1 cytokine family and encompass the pro-inflammatory cytokines IL-36α, IL-36β and IL-36γ, as well as the anti-inflammatory cytokine IL-36Ra (1). Each of the IL-36 cytokines is encoded by individual genes, which in humans are located on chromosome 2 in between the *il1b* and *il1br* loci (2–4), and share sequence and structural homology. All members of the IL-36 subfamily were identified and partly characterized in the early 2000’s by different research groups, and consequently, they were initially referred to by different names (5–8) (**Table 1**). To avoid confusion, a new nomenclature system was adopted in 2001 (10). In this system, the IL-36 cytokines were designated as IL-1F5, IL-1F6, IL-1F8 and IL-1F9 based on their relationship to the IL-1 cytokine family and the order of their date of publication (10) (**Table 1**). In 2010, the current nomenclature was adopted, from which IL-1F5 was designated as IL-36Ra, IL-1F6 was designated as IL-36α, IL-1F8 was designated as IL-36β, and IL-1F9 was designated as IL-36γ (9) (**Table 1**).

**Table 1:** Current and former nomenclature of IL-36 cytokines.

Current nomenclature (9)	First unified nomenclature (10)	IL-1 extended nomenclature (8)	Family of IL-1 (FIL1) based nomenclature (7)	IL-1-related protein (IL1RP) based nomenclature (6)	IL-1 homologues (IL-1H) based nomenclature (5)	Role in inflammation
IL-36α	IL1-F6		FIL1ϵ			Pro-inflammatory
IL-36β	IL1-F8		FIL1η		IL-1H2	Pro-inflammatory
IL-36γ	IL1-F9	IL-1ϵ		IL-1RP2	IL-1H1	Pro-inflammatory
IL-36Ra	IL1-F5	IL-1δ	FIL-1δ	IL-1RP3	IL-1H3	Anti-inflammatory

After the discovery of this subfamily of cytokines, functional studies, aimed to understand the biology of IL-36 cytokines, as well as their role in inflammation were performed using *in vitro* platforms. The first functional analysis was restricted to IL-36γ (initially referred to as IL-1ϵ) and IL-36Ra (referred to as IL-1δ) (8). This first study determined that IL-36γ enhances the activation of Nuclear Factor kappa-light-chain-enhancer of activated B cells (NF-κB) signaling through the orphan receptor IL-1 Receptor-Related protein 2 (IL-1Rrp2) – a receptor that is highly expressed in epithelial cells and in embryonic tissue. However, IL-36Ra inhibits NF-κB activation induced by IL-36γ (8). Subsequently, it was shown that the activation of the NF-κB pathway mediated by IL-36γ requires the accessory protein Interleukin-1 receptor accessory protein (IL-1RAcP) (11), which is vital for IL-1 signaling (12). The requirement of IL-36R and IL-1RAcP for NF-κB activation was later described also for IL-36α and IL-36β (11). These initial studies also provided the first mechanistic insights regarding the antagonist effect of IL-36Ra, which competes with IL-36α, IL-36β, and IL-36γ for the IL-1Rrp2 binding pocket without inducing the recruitment of IL-1RAcP and therefore preventing NF-κB signaling (8). Mechanistically, this inhibitory process is similar to the inhibition of IL-1 signaling mediated by IL-1Ra (13) that is discussed in detail in the following sections.

Despite the ability of IL-36 cytokines to activate NF-κB signaling through IL-1Rrp2/IL-1RAcP, controversy existed regarding their biological functions during physiological processes. This was due, in part, to the fact that the concentration of IL-36γ required to observe a biological effect *in vitro* varied considerably among reports (50 ng/ml in Debets et al., 2001 vs 500 ng/ml in Towne et al., 2004), and because the antagonistic effects of IL-36Ra could not be uniformly reproduced (8, 11). It is now established that, unlike other IL-1 family members that possess caspase cleavage sites, the IL-36 cytokines do not (1) and require post-translational processing at the N-terminal region in order to be fully active (14). N-terminal processing of IL-36α, IL-36β, IL-36γ and IL-36Ra proximal to K^6^, R^5^, S^18^ and V^2^, respectively, leads to the enhancement of their biological activity (1,000-10,000 fold) (14). The N-terminal processing of IL-36 cytokines provided a logical explanation for some of the earlier controversies regarding IL-36 cytokine function and highlights the complexity of their biology.

As IL-1Rrp2 is highly expressed in the skin, considerable research has focused on understanding the role of IL-36 cytokines in the context of skin diseases, such as psoriasis and dermatitis (11, 15). However, IL-1Rrp2 and IL-36 cytokines are present in other tissues, including the lungs (16), which is an organ continuously exposed to a wide variety of pathogens, antigens and noxious agents that can lead to unwarranted inflammation if not properly regulated. In this review, we describe the factors that trigger the production and activation of IL-36 cytokines, as well as the inflammatory effects of IL-36 cytokines in immune cells. We then discuss the central role of IL-36 cytokines in regulating the host immune response during lung infection and other inflammatory diseases, and finally conclude with current therapeutic tools in clinical trials designed to target the IL-36 pathway and their potential use during lung inflammation.

## Production of IL-36 Cytokines Is Mediated by Toll-Like Receptor and Pro-Inflammatory Cytokine Signaling

**Figure 1:**
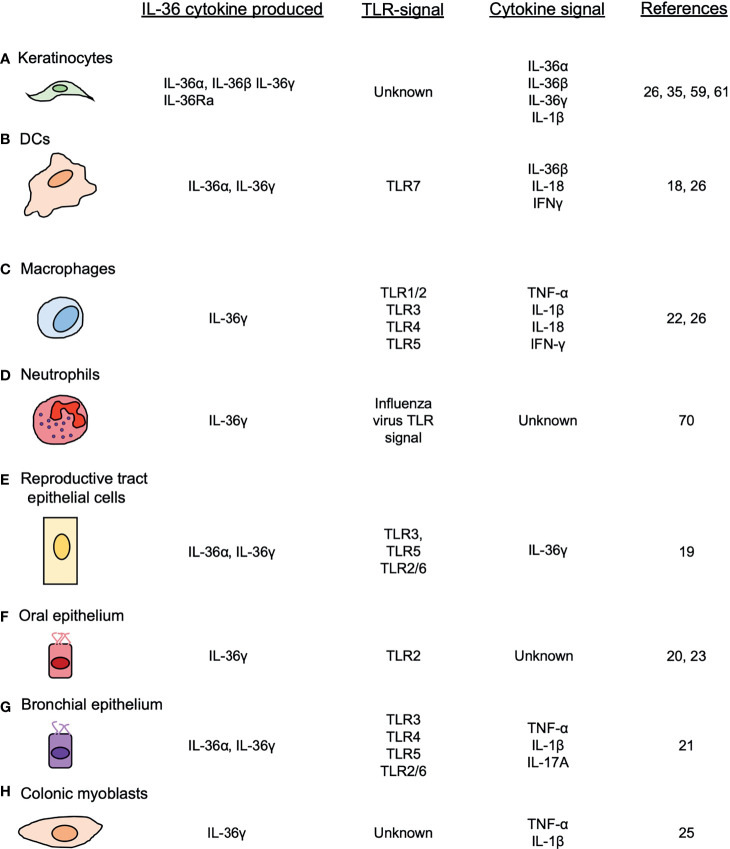
Cellular sources of IL-36 cytokines. IL-36 cytokines are produced by different cell types, such as **(A)** keratinocytes, **(B)** DCs, **(C)** macrophages **(D)** neutrophils, **(E)** female reproductive epithelial cells, **(F)** oral epithelial cells **(G)** bronchial epithelial cells and **(H)** colonic myoblasts, after the induction of TLRs and other pro-inflammatory cytokines.

**Figure 2:**
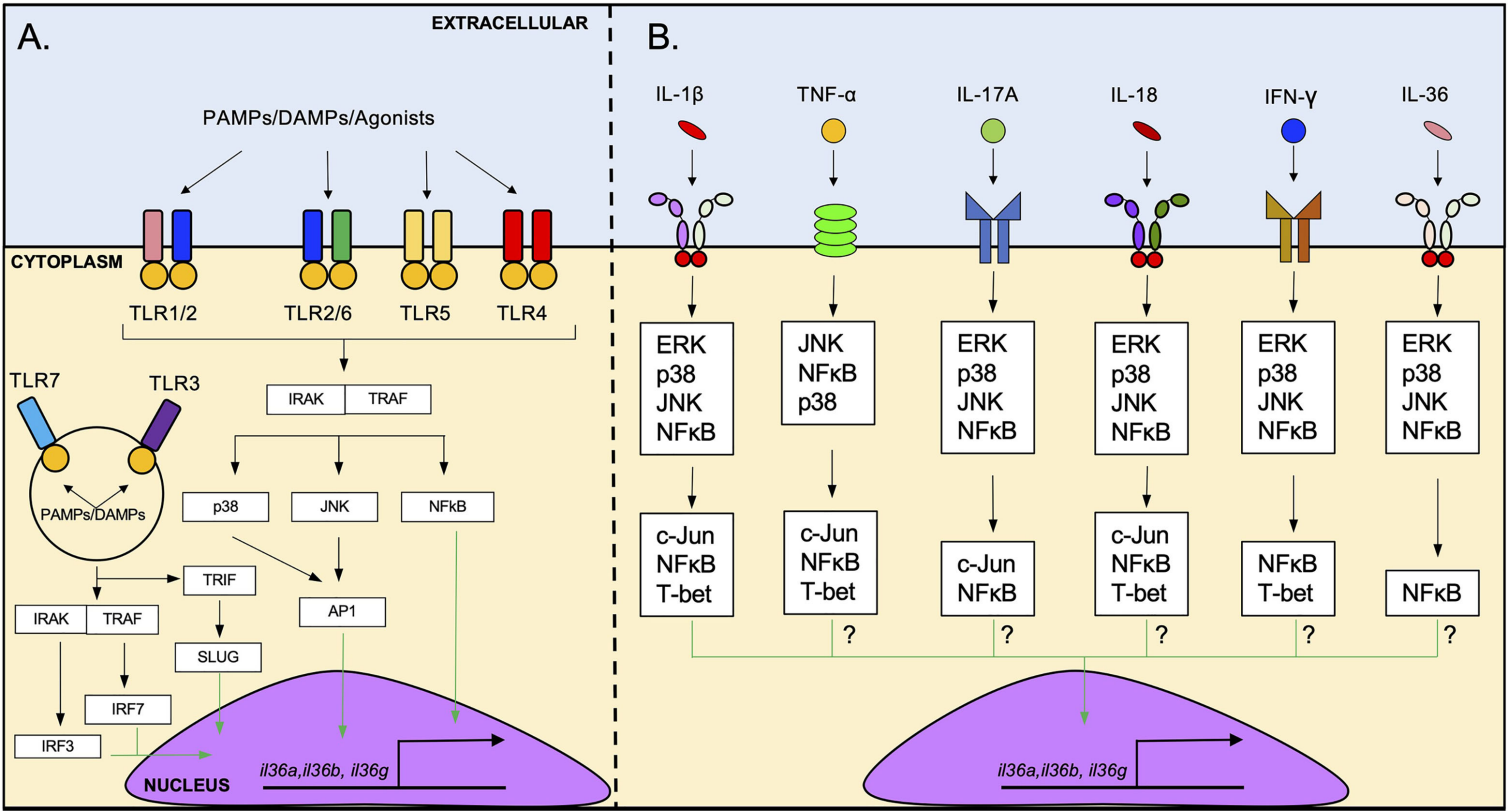
Induction of IL-36 cytokine expression by TLR agonists and pro-inflammatory cytokines. **(A)** *il36* transcription is triggered by the activation of several cell surface TLRs such as TLR1/2, TLR2/6, TLR5 and TLR4. The possible intracellular pathway that induces *il36* transcription downstream of TLRs activation involves signaling *via* IRAK/TRAF and p38/JNK/NFκB, followed by the translocation of AP1 and NFκB to the nucleus. Intracellular TLRs such as TLR3 and TLR7 can also induce *il36* transcription. These receptors can activate IRF3/7 which may induce the transcription of *il36*. **(B)** *il36* expression is induced by several pro-inflammatory cytokines such as IL-1β, TNF-α, IL-17A, IL-18, IFN-γ and IL-36 cytokines. IL-1β-mediated induction of *il36* expression *via* IL1R-IL1AcP involves the ERK/p38/JNK/NFκB signaling and subsequent activation of the transcription factors c-Jun, T-bet and NFκB. The exact pathway *via* which other cytokines induce *il36* transcription has not been determined yet but likely involves similar intracellular signaling molecules.

Several studies have provided insights regarding the cellular sources **(** **Figure 1** **)** and signals **(** **Figure 2** **)** that induce the production of IL-36 cytokines, particularly IL-36α and IL-36γ. Like other pro-inflammatory cytokines, the production and activity of IL-36 cytokines during inflammatory processes are tightly regulated. Signaling through a variety of toll-like receptors (TLRs) has been shown to induce IL-36 production **(** **Figure 2A** **)**. For example, an *in vivo* model of acute skin injury demonstrated that TLR-3 signaling is required for IL-36γ expression by keratinocytes. The induction of IL-36γ by TLR-3 is mediated by TRIF and involves the activation of the transcription factor SLUG and the subsequent inhibition of the vitamin D receptor (VRD) (17).

The TLR7 agonist Imiquimod is a potent inducer of IL-36α and IL-36γ in bone-marrow-derived dendritic cells (BMDCs) *in vitro* and in a model of dermatitis *in vivo* (18). In this study, Imiquimod induced the *in vitro* production of IL-36α and IL-36γ by BMDCs and the specific depletion of CD11c cells ameliorated the cutaneous pathology *in vivo* (18). Another study showed that cultured human female reproductive tract epithelial cells produced IL-36γ after treatment with different TLR ligands, including Poly(I:C) (TLR3), Flagellin (TLR5) and FSL-1 (TLR2/6) (19). Others have reported that nasal epithelial cells produce IL-36γ *via* TLR2/IRAK/IRF6 in response to the Gram-negative bacterium *Porphyromonas gingivalis* (20). Relevant to the lungs, *in vitro* studies using primary cells and cell lines have shown that both pulmonary macrophages and bronchial epithelial cells produce IL-36α and IL-36γ, but not IL-36β or IL-36Ra in response to different TLR agonists (21, 22). Interestingly, whereas PAM_3_CSK_4_ (TLR1/2), Poly(I:C) (TLR3), LPS (TLR4) and Flagellin (TLR5) were shown to induce *il36g* expression in pulmonary macrophages (22), dsRNA (TLR3), LPS (TLR4), Flagellin (TLR5) and FSL-1 (TLR2/6) induced the expression of *il36a* and *il36g* in cultured human bronchial epithelial cells. These data show that TLR ligands induce the expression of *il36* genes in different cell types, although it also suggests that the induction of specific *il36* genes varies depending on the ligand as well as the cellular context (21).

During *Candida albicans* infection, IL-36 cytokines production by human oral epithelial cells (TR146 cells) is dependent upon p38, NF-κB and PI3K signaling, as their inhibition, significantly diminished the expression of Il36a and Il36g (23). Although this study did not provide insights regarding which TLR triggers the production of IL-36 by these cells, TLR2 and TLR4 signaling may induce the production of IL-36 cytokines in this model (24).

Besides TLR signaling, several pro-inflammatory cytokines can induce IL-36 cytokine production through the activation of their cognate receptors **(** **Figure 2B** **)**. For example, IL-1β and TNF-α have been identified as potent inducers of IL-36γ in human colonic myoblasts (25) and in mouse pulmonary macrophages (22). IL-1β, TNF-α and IL-17A are potent inducers of IL-36α and IL-36γ in human bronchial epithelial cells, acting synergistically with each other to induce a robust response (21). IL-18 and IFN-γ induce the production of IL-36γ in the cell line KG-1 – that resembles human dendritic cells (DCs) – and primary human DCs and macrophages (26). In addition to these cytokines, several reports have also described the ability of IL-36 cytokines to enhance their own expression. For example, BMDCs stimulated with IL-36β increased expression of *il36a* and *il36g* (18). Similarly, treatment of HaCat cells (human immortalized keratinocytes) with IL-36γ has been shown to increase *il36g* expression (26), and IL-36γ treatment of vaginal and endocervical epithelial cells leads to increased IL-36γ production *in vitro* (19). Mechanistically, the induction of IL-36γ by IL-1β appears to involve ERK1/2, JNK1/2, p38, c-Jun and NF-κB signaling, as inhibition of these signaling pathways reduced *il36g* expression in human colonic myoblasts (25) **(** **Figure 2B** **)**. TNF-α (27), IL-17A (28), IL-18 (29, 30), IFN-γ (31) and IL-36 cytokines (29, 30) are known to activate MAPK signaling and NF-κB signaling **(** **Figure 2B** **)**, suggesting that these cytokines may trigger IL-36 production in a similar fashion as IL-1β.

The transcription factor T-bet has been shown to be positively involved in *il36g* expression in myeloid cells, by binding to a T-box motif present in the position -500 of the *il36g* promoter (26). T-bet knockdown significantly reduced the expression of *il36g* in response to IFN-γ, IL-1β and TNF-α in these cells (26). T-bet is expressed in lymphoid and myeloid cells (32). In T cells, T-bet expression is required for the development of a Th1 response (33). Mechanistically, T-bet induces a Th1 response through the recruitment of the super elongation complex (SEC), positive transcription elongation factor (P-TEFb) and the multiprotein complex called “Mediator”, all of which are required for transcription of Th1 genes (34). NF-κB has also been reported to recruit this same complex, albeit through a different molecular mechanism than T-bet (34). Whether NF-κB and T-bet cooperate with each other to induce the transcription of *il36g* and potentially other IL-36 cytokines in myeloid cells is unknown and requires further study. Another transcription factor involved in *il36g* expression is Nuclear factor erythroid 2 (Nrf2). Specifically, Nrf2 overexpression increased *il36g gene* expression and IL-36γ protein production in the epidermis in a murine model. The pharmacological activation of Nrf2 on murine immortalized keratinocytes (MIKs) and HaCaT cells treated with tert-butylhydroquinone (tBHQ) also increased the expression of *il36g* (35). Mechanistically, this study found that Nrf2 binds to three Antioxidant Response Elements (ARE) enhancers located upstream to the putative transcriptional start site of *il36g*, thereby inducing *il36g* expression (35).

The identification of the intracellular adaptors involved in the induction of IL-36 cytokines by IL-1β (25) provides a logical framework for understanding the downstream signaling pathways involved in the induction of IL-36 cytokines by other pro-inflammatory cytokines. For example, the downstream signaling pathways activated by IL-18 and IL-36 cytokines, both members of the IL-1 family, are similar to the ones activated by IL-1β (29, 30) **(** **Figure 2B** **)**. TNF-α, IL-17A and IFN-γ signaling also share important downstream signaling pathways with IL-1β signaling that mediate the induction of IL-36 cytokines, including JNK, ERK, p38 and NF-κB (27, 28, 31) **(** **Figure 2B** **)**. These observations suggest overlap of the different signaling pathways and some redundancy in the system that mediate the production of IL-36 cytokines in response to different cytokine signals **(** **Figure 2B** **).**

## Neutrophil, Macrophage/Epithelial and Microbe-Derived Proteases Process IL-36 Cytokines

**Figure 3:**
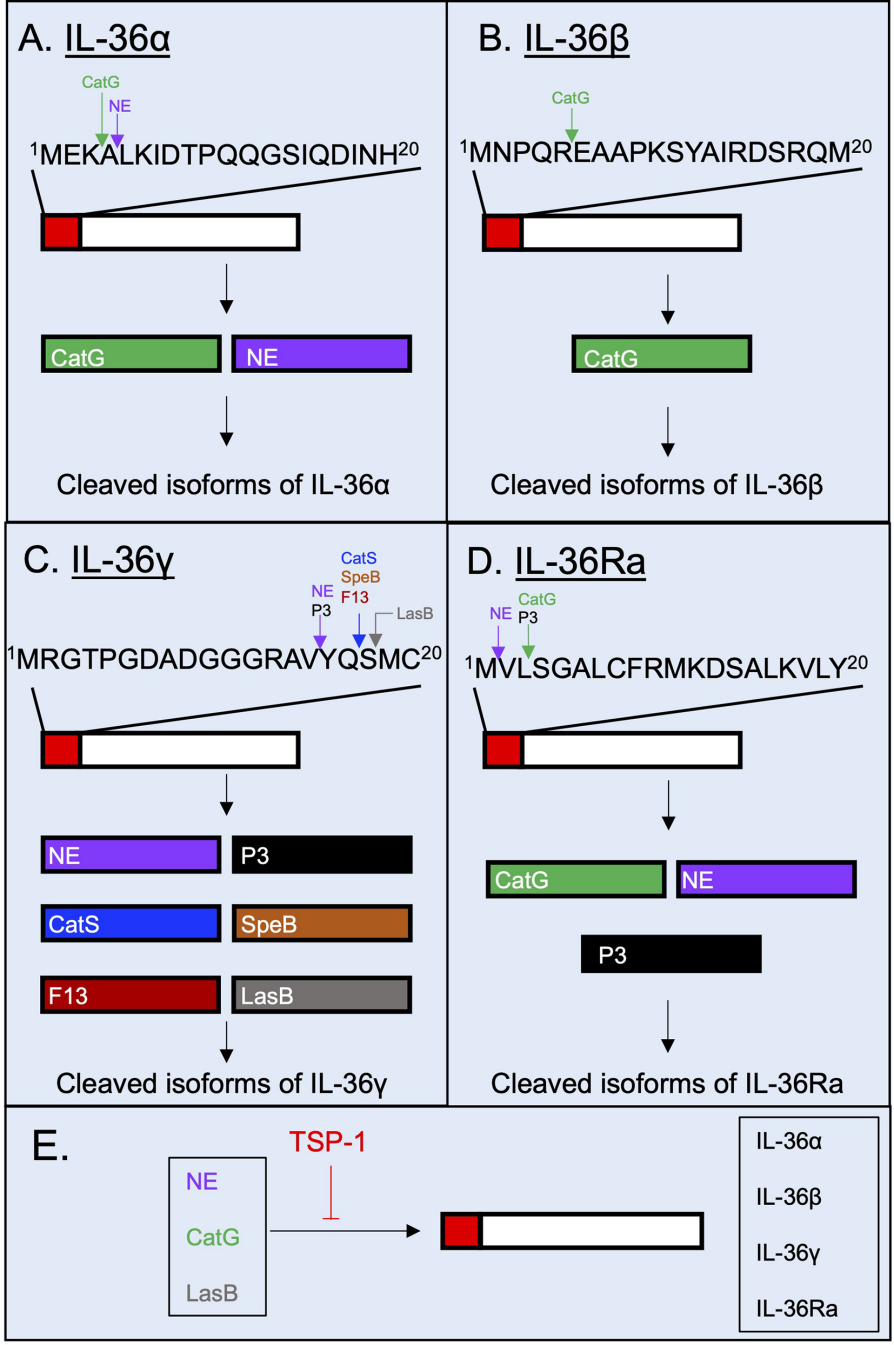
Post-translational processing of human IL-36 cytokines by extracellular proteases. Human IL-36 cytokines are selectively cleaved and potentially activated by proteases. **(A)** IL-36α is cleaved extracellularly by neutrophil proteases Cathepsin G (CatG) and Neutrophil Elastase (NE). **(B)** IL-36β has been described to be N-terminally processed by CatG. **(C)** IL-36γ is selectively cleaved by Proteinase-3 (P3), NE, Cathepsin S (CatS), the *Streptococcus pyogenes*-derived protease SpeB, the *Aspergillus fumigatus*-derived protease F13 and the *Pseudomonas aeruginosa*-derived protease LasB. **(D)** IL-36Ra is cleaved and potentially activated by NE, CatG and P3. **(E)** The proteolytic cleavage of IL-36 cytokines can be regulated by the host glycoprotein thrombospondin-1, which has been shown to possess inhibitory properties against the host proteases NE and CatG, and against the bacterial protease LasB.

In order to be fully active, IL-36 cytokines undergoes N-terminal processing by proteases (14). It was unclear for some time which proteases were responsible for processing and activating IL-36 cytokines. However, in 2016, a study showed that the supernatant of PMA-activated neutrophils cleaved IL-36α, IL-36β and IL-36γ, and induced the production of pro-inflammatory cytokines and chemokines in IL-1Rrp2-transfected HeLa cells (36). These observations suggested that neutrophil-derived proteases can cleave and activate IL-36 cytokines. The incubation of different recombinant neutrophil-derived proteases with each IL-36 cytokine and the subsequent incubation of the cleaved products with IL-1Rrp2-transfected HeLa cells led to the identification of different neutrophil-derived proteases that can post-translationally process IL-36 cytokines. Whereas IL-36α is cleaved proximal to A^4^ and L^5^ by Cathepsin G (CatG) and neutrophil elastase (NE), respectively (36, 37) **(** **Figure 3A** **)**, IL-36β is cleaved proximal to E^6^ by CatG **(** **Figure 3B** **)**, and IL-36γ can be cleaved proximal to Y^16^ by either NE and proteinase-3 (P3) (36, 37) **(** **Figure 3C** **)**. In these cases, cleavage leads to increased biological activity of the IL-36 cytokine in IL-1Rrp2 transfected HeLa cells and in primary keratinocytes, although possible differences in the activity of each of these cleaved products have not been evaluated (36). Like the pro-inflammatory IL-36 cytokines, the activity of the anti-inflammatory IL-36Ra is also regulated by proteolytic processing (38). IL-36Ra can be activated following cleavage proximal to V^2^ by NE or through cleavage proximal to S^4^ by CatG and P3 (38) **(** **Figure 3D** **)**. Importantly, the activation of IL-36 cytokines does not appear to be limited to neutrophil proteases. Cathepsin S (CatS) cleaves IL-36γ proximal to S^18^ and the cleavage product has been described as the most bioactive form of IL-36γ (39) **(** **Figure 3C** **)**. In this study, CatS, expressed by keratinocytes and fibroblasts, was highly upregulated in response to treatment with various pro-inflammatory cytokines, including TNF-α, IFN-γ, IL-17 and IL-22 (39). This study showed that the Y^16^ isoform produced by NE failed to induce the production of IL-8 in HaCaT cells *in vitro* when compared to the S^18^ isoform (39). However, this study also demonstrated that the incubation of full-length IL-36γ in the presence of increasing molar concentrations of neutrophil proteases, increased IL-8 secretion by HaCaT cells albeit to a lesser effect when compared to CatS (39). While the IL-36γ cleaved just proximal to Y^16^ does not display the same bioactivity as IL-36γ cleaved proximal to S^18^ *in vitro*, neutrophil elastase appears to induce the production of IL-8 in HaCaT cells when co-incubated with full length IL-36γ. This conclusion is supported by at least three studies that show that neutrophil serine proteases cleave and increase the bioactivity of IL-36 cytokines (36, 37, 40). In these studies, full-length IL-36α, IL-36β and IL-36γ induced IL-6, IL-8 and CXCL1 production by transfected IL-36R^+^ Hela cells when supernatant from PMA-activated neutrophils was added, and this effect was diminished when cathepsin G and elastase inhibitors were included (36, 37, 40). Further *in vitro* studies using purified cathepsin-G, neutrophil elastase and proteinase-3 determined that neutrophil proteases induce the bioactivity of the different IL-36 cytokines as well as identification of the cleaved isoforms. Importantly, none of the studies that have determined the N-terminal processing by neutrophil proteases (36, 37, 39–41) have identified the S^18^ isoform of IL-36γ as a cleaved product. Further *in vivo* studies that capture the complexity of the biology surrounding the production and activation of IL-36 cytokines are required to accurately conclude whether IL-36 isoforms generated by neutrophil proteases amplify inflammation in *in vivo* models.

CatS is not only expressed by keratinocytes, but also by lung epithelial cells (42), intestinal epithelial cells (43) and human monocytic THP-1 cell line (44). Importantly, a recent study has demonstrated that pathogen-derived proteases secreted by *Streptococcus pyogenes* (SpeB), *Aspergillus fumigatus* (Aspergillus factor 13 – F13), as well as unknown proteases produced by *Staphylococcus aureus* and *Trichophyton rubrum* can process IL-36γ proximal to S^18^ **(** **Figure 3C** **)** (45). Moreover, in a recent study, we demonstrated that the *Pseudomonas aeruginosa* elastase B (LasB), cleaves IL-36γ proximal to M^19^, just one residue after S^18^ which is the site of cleavage mediated by CatS, SpeB and F13 (41) **(** **Figure 3C** **)**. *In silico* docking analysis predicted similar binding of the M^19^ isoform to the IL-1Rrp2/IL-1RacP heterodimer compared to the S^18^ isoform, although the biological activity of M^19^ isoform remains to be experimentally confirmed (41). Our work also showed that extracellular processing of IL-36 cytokines is counter-balanced by protease inhibitors released by a variety of cells during inflammatory processes such as the host matricellular glycoprotein thrombospondin-1 (TSP-1) which regulates the proteolytic activity of NE, CatG and LasB, and thereby dampening IL-36γ-mediated inflammation (41, 46, 47) **(** **Figure 3E** **)**.

## IL-36 Cytokines Are Recognized by IL-1Rrp2/IL-1RAcP and Trigger Several Inflammatory Processes That Amplify Host Inflammation

### IL-1Rrp2 Expression, Receptor-Ligand Interactions and Intracellular Signaling

Once IL-36 cytokines are cleaved and fully activated, they bind to IL-1Rrp2 and transduce a complex signaling cascade that turns on an inflammatory response (48). In humans, IL-1Rrp2 is widely expressed in keratinocytes in the skin and the esophagus but poorly expressed in other organs like reproductive organs, lymph nodes or lungs (16). Various immune cells also express IL-1Rrp2 (49–52). One report showed that circulating DCs and monocytes, but not CD3^+^ T cells express IL-1Rrp2 on their surface and respond to IL-36 cytokines *in vitro* (49). A subsequent study showed that CD19^+^ B cells and CD8^+^ T cells express IL-1Rrp2 on their surface but CD4^+^ T cells expressed high amounts of IL-1Rrp2 in the cytoplasm (50). Human neutrophils do not express IL-1Rrp2 at baseline (51), but during chronic rhinosinusitis where IL-36 cytokines are locally upregulated, neutrophils were found to be the main cell type expressing IL-1Rrp2 in nasal polyps, but not in the bloodstream (51). This finding suggests that the microenvironment can induce the expression of IL-1Rrp2 in neutrophils that normally do not express this receptor. This hypothesis is supported by the fact that peripheral blood neutrophils, which do not express IL-1Rrp2 under basal conditions, expressed IL-1Rrp2 after incubation with the pro-inflammatory cytokines IL-6 and IL-1β (51). In mice, the expression of IL-1Rrp2 in immune cells follows a similar pattern as in humans with some notable differences. BMDCs, generated from bone marrow precursors in presence of GM-CSF, and CD4^+^ T cells express IL-1Rrp2 and can respond to IL-36 agonists in the absence of any other stimuli (52). Neutrophils and macrophages derived from the bone marrow (BMDMs), on the other hand, do not significantly express IL-1Rrp2 at baseline (52).

**Figure 4:**
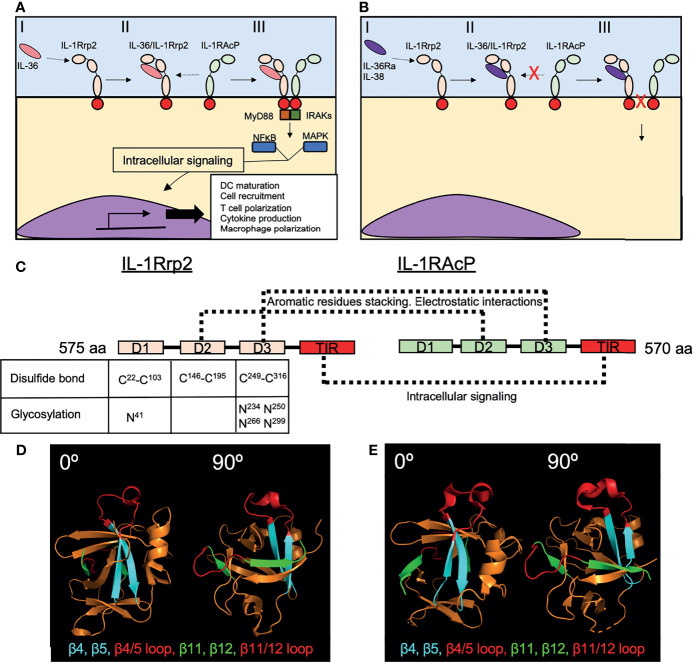
Recognition of IL-36 cytokines by the IL-1Rrp2/IL-1RAcP complex. **(A)** The initial recognition of IL-36 agonists by IL-1Rrp2 on the surface of the cell (I), triggers the recruitment of IL-1RAcP to the receptor-ligand complex (II), initiating an intracellular pathway characterized by the activation of MyD88 and IRAKs and then NFκB and MAPK that will induce the transcription of several genes involved in DC maturation, T cell and macrophage polarization and cytokine production (III). **(B)** IL-36Ra and IL-38 also bind IL-1Rrp2 (I) but this binding does not recruit IL-1RAcP (II), thus inhibiting the inflammatory response triggered by IL-36 agonists (III). **(C)** IL-1Rrp2 and IL-1RAcP are composed of three extracellular domains (D1-D3) and one TIR intracellular domain. Disulfide bonds and N glycosylation found in D1, D2 and D3 of IL-1Rrp2 are critical for IL-1Rrp2/IL-36 binding. Direct interactions between IL-1Rrp2 and IL-1RAcP through their respective D2s and D3s have been identified in absence of IL-36 agonists. **(D)** Loops between β4/β5 and β11/β12 present in human IL-36γ (PDB 4IZE) mediate interaction with IL-1RAcP. **(E)** Mouse IL-36Ra (PBD 4P0L) has similar loops in these regions with a slightly different structural conformation that avoid the interaction with IL-1RAcP. Crystal structures were obtained from the Protein Data Bank (https://www.rcsb.org) and modeled in PyMOL (2.3.3).

The molecular mechanism by which IL-36 cytokines bind to IL-1Rrp2 is similar to the mechanism described for IL-1/IL-1R (53). The IL-36 receptor heterodimer is comprised of IL1-Rrp2 and the accessory protein IL-1RAcP (54). When agonistic IL-36 cytokines bind to IL-1Rrp2 on the cell surface, IL-1RAcP is recruited to the IL-36 receptor-ligand complex (54). Once the complex is complete, TIR domains present within the intracellular region of IL-1Rrp2 and IL-1RAcP recruit MyD88 and IL-1R associated kinase (IRAK), which in turn trigger NF-κB and MAPK activation to promote the expression of pro-inflammatory genes **(** **Figure 4A** **)**.

The anti-inflammatory cytokine, IL-36Ra (K*_D_* =5.8nM) binds with a higher affinity to IL-1Rrp2 compared to N-terminally processed K^6^-IL-36α (K*_D_* =480nM) and S^18^-IL-36γ (K*_D_* =1800nM) (54). A previous study analyzed the affinity of the IL-36 cytokines that bind to the IL-1Rrp2/IL-1RAcP heterodimer. This study found that K^6^-IL-36α (K*_D_* =0.021nM), R^5^-IL-36β (K*_D_* =0.007nM) and S^18^-IL-36γ (K_D_=0.147nM) bound to the IL-1Rrp2/IL-1RAcP heterodimer with higher affinity than V^2^-IL-36Ra (K_D_=10.2nM) (14). From a biological standpoint, IL-36 agonists initially bind to IL-1Rrp2, promoting the recruitment of IL-1RAcP that leads to subsequent signaling. Whereas the first study showed that the dominant pathway of IL-36 activation is through agonist-ILRrp2 complex formation and subsequent recruitment of IL-1RAcP, IL-1RAcP does not bind IL-1Rrp2 when no agonist is present (54). In contrast to agonist IL-36 cytokines, the binding of IL-36Ra to IL-1Rrp2 does not result in the recruitment IL-1RAcP, thus preventing the induction of downstream signaling (54) **(** **Figure 4B** **)**. While the Towne et al., 2011 findings show that the affinity of processed IL-36 agonists are in orders of magnitude higher than IL-36Ra, the findings focused on binding affinity to the heterodimer which is restricted to the agonists and not IL-36Ra. In other words, IL-36Ra does not bind the heterodimer but just binds to IL-1Rrp2.

IL-38, another cytokine from the IL-1 family, also binds to IL-36R and suppresses the pro-inflammatory activity of IL-36 cytokines through a similar mechanism as IL-36Ra (55) **(** **Figure 4B** **)**. Although the crystal structure of IL-36R has not been resolved, biochemical and *in silico* approaches have provided important insights regarding IL-1Rrp2-ligand interactions (54, 56, 57). IL-1Rrp2 consist of three extracellular structural domains (D1-D3) and one intracellular TIR domain that triggers downstream signaling **(** **Figure 4C** **)**. The three extracellular domains are part of the ligand-binding pocket, where D1 conforms to the upper part of the pocket, D2 the backside and D3 the bottom part (56). D2 of IL-1Rrp2 is also important for the interaction with IL-1RAcP (56). Disulfide bonds between Cysteine residues C^22^/C^103^, C^146^/C^195^ and C^249^/C^316^ in D1, D2 and D3 respectively as well as Asparagine (N) linked glycosylation of IL-1Rrp2 at positions N^41^ (D1), N^234^, N^250^, N^266^, N^299^ (D3) of the extracellular domains are required for the intracellular signaling and trafficking to the cell surface of IL-1Rrp2 **(** **Figure 4C** **)**. Molecular modeling predicted the relevance of these IL-1Rrp2 sites and confirmed biochemically after mutating each cysteine or asparagine to alanine. Each of these substitutions impaired IL-1Rrp2/IL-1RAcP signaling in response to agonist (56).

Different residues within the structural domains were found to be crucial for IL-36α, IL-36β and IL-36γ to bind and induce signaling *via* IL-1Rrp2/IL-1RAcP, suggesting that each IL-36 cytokine is recognized differently by IL-1Rrp2 (56). This hypothesis has been corroborated by testing the role of specific amino acids (N^41^, C^42^ and C^118^) of IL-1Rrp2 in response to each IL-36 cytokine (56). Whereas substitution of N^41^ by A impaired IL-1Rrp2-mediated signaling in response to the three pro-inflammatory IL-36 cytokines, C^42^ substitution highly impaired the response to IL-36α but did not affect the IL-36β response and only mildly affected the response to IL-36γ. Similarly, substitution of C^118^ highly affected IL-36α-responsiveness, but not IL-36γ responsiveness and only mildly affected IL-36β-responsiveness (56). Regarding why the binding of pro-inflammatory IL-36 cytokines to IL-1Rrp2 induces the recruitment of IL-1ARcP whereas the ligation of IL-36Ra to IL-1Rrp2 does not, one report identified the existence of two structural loops present in human IL-36γ – one between β-strands 11 and 12 and one between β-strands 4-5 – that appear to be involved in the recruitment of IL-1RAcP to the IL-1Rrp2/IL-36γ complex and the consequent induction of downstream signaling **(** **Figure 4D** **)** (57). Similar loops are also present within IL-36Ra, but these loops slightly differ in structure from those observed in IL-36γ **(** **Figure 4E** **)**. Interestingly, the exchange of these loops between IL-36γ and IL-36Ra inverted the ability of these cytokines to bind IL-1RAcP (57), indicating that these structural regions are crucial for the recruitment of IL-1RAcP to the receptor-ligand complex. IL-36γ directly interacts with both IL-1Rrp2 and IL-1RAcP (56), and thus, IL-36γ may facilitate the recruitment of IL-1RAcP to the IL-36 receptor-ligand complex. However, molecular modeling and biochemical approaches also established a direct interaction between IL-1Rrp2 and IL-1RAcP in the absence of a ligand (56). This interaction involves aromatic residues stacking and electrostatic interactions between D2-D2 and D3-D3 interactions of both receptors (56)** (** **Figure 4C** **)**.

### Pro-Inflammatory Effects of IL-36 Signal in Lymphoid and Myeloid Cells

**Figure 5:**
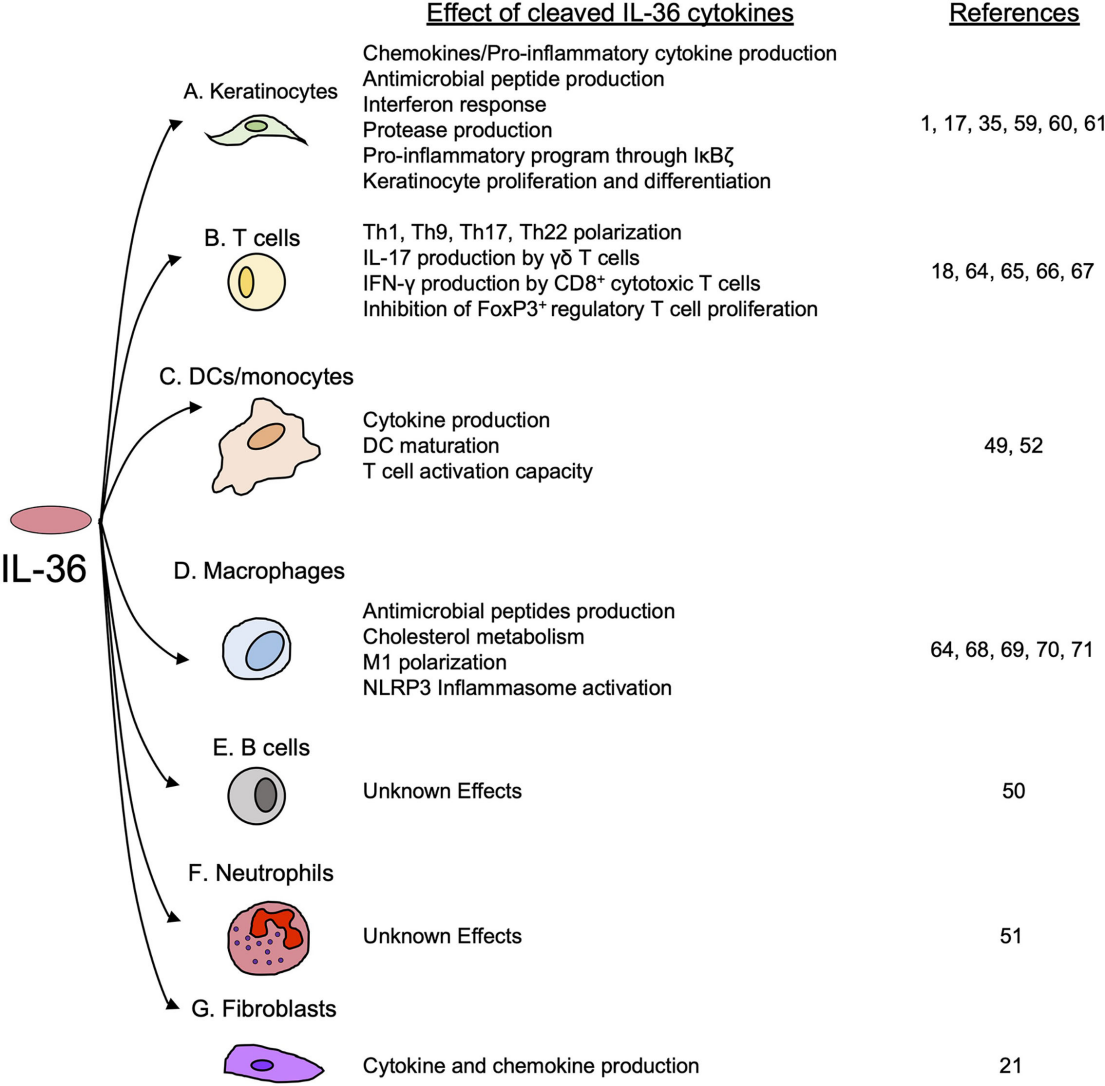
The effects of cleaved IL-36 cytokines on keratinocytes and immune cells. IL-36 cytokines trigger the activation of several inflammatory processes in **(A)** keratinocytes and various immune cells, including **(B)** T cells, **(C)** DCs and monocytes and **(D)** macrophages. **(E)** B cells and **(F)** neutrophils are known to express IL-1Rrp2, but the effects of IL-36 cytokines on their function have not yet been determined. Finally, IL-36 cytokines have shown to induce the production of pro-inflammatory cytokines in **(G)** fibroblasts.

Even though IL-36 cytokines are not directly chemotactic to inflammatory/immune cells, they strongly triggers pro-inflammatory cytokine/chemokine production *via* MAPK- and NF-κB-signaling in a variety of cells and tissues (58). As IL-1Rrp2 is highly expressed in keratinocytes, many studies investigating the biological effects of IL-36 cytokines have initially focused on their role during skin inflammatory disorders such as psoriasis and dermatitis. In keratinocytes, IL-36 cytokines enhance the production of a variety of pro-inflammatory cytokines and chemokines, including CXCL-8, IL-23A, IL-6, IL-8 and TNF-α, as well as antimicrobial peptides such as β-defensins 2 and 3, the human cathelicidin LL37 and the antimicrobial psoriasin S10047 (1, 59, 60) **(** **Figure 5A** **)**. It has been shown that, in keratinocytes, IL-36 cytokines enhance their own production in an autocrine manner (35, 61). Interestingly, in keratinocytes, IL-36 cytokines also induce the production of IL-36Ra (59), which may be a crucial negative feedback mechanism to regulate the inflammatory effects of IL-36 cytokines.

Of particular interest is the observation that IL-36α and IL-36γ induce the expression of NF-κB inhibitor zeta (IκBζ) in keratinocytes (60). IκBζ is a transcription factor that regulates some NF-κB target genes in response to IL-36α and IL-36γ (60). In primary keratinocytes and HaCaT cells, IκBζ is essential for IL-36-induced inflammation, and in the context of IL-36 stimulation, its silencing downregulates several genes involved in a variety of processes, including neutrophil chemotaxis, IL-6 response factors, STAT phosphorylation, leukocyte adhesion and differentiation, T cell-activation, innate immunity and leukocyte activation (60) **(** **Figure 5A** **)**. Moreover, it has been described that during injury, IL-36γ induces keratinocyte differentiation and proliferation through the induction of Reg3A (17) **(** **Figure 5A** **)**.

Although IL-1Rrp2 expression is generally highest in the skin compared to other organs and tissues (16), IL-36 cytokines also exert important inflammatory effects in other tissues. In the intestine, it has been described that IL-36 cytokines induce and amplify the expression of the IL-23/IL-22 axis (62), as IL-36R^-/-^ mice with DSS-induced colitis present with reduced expression of IL-23 and IL-22 in the intestine compared to WT mice (62). Ex vivo analysis of colonic explants from DSS treated mice showed that IL-36γ induced the expression of IL-23 in DCs through a Notch2-dependent mechanism. The induction of IL-23 by IL-36γ was found to be mediated by NF-κB and promoted the recovery of colonic damage induced by DSS *via* the induction of IL-22 responses (62). This IL-36/IL-23/IL-22 axis has been recently proven to be important in host defense against the enteropathogenic bacteria *Citrobacter rodentium* (63). In this model of infection, IL-36γ induces the production of IL-22 in CD4^+^ T cells in the presence of DCs, a response that is mediated by the transcription factor Aryl hydrocarbon receptor (AhR) and by the production of IL-6 (63). Here, the activity of IL-36γ is protective against *C. rodentium* infection, as IL-36R^-/-^ mice not only showed impaired bacterial clearance and higher intestinal damage, but also impaired survival compared to WT mice (63).

Another major effect of IL-36 cytokines is the activation of the IL-23/IL-17A axis in the skin and in the kidney during inflammation and fibrosis (18, 64). In a model of psoriasiform dermatitis, IL-36 cytokines produced by DCs were shown to induce the recruitment of neutrophils and γδ T cells (18). In this model, lack of IL-1Rrp2 reduces the disease severity and at the same time reduces the production of IL-17A by γδ T cells (18). IL-17A^-/-^ and IL-23^-/-^ mice showed the same phenotype, suggesting that IL-36 cytokines contribute to the severity of experimental psoriasiform dermatitis by inducing the expression of IL-23 and IL-17A (18).

Interestingly, IL-36α expression is also increased in chronic kidney disease in patients with renal tubulointerstitial lesions (64). In a mouse model of unilateral ureteral obstruction that mimics the renal tubulointerstitial lesions observed in humans, IL-36α activated the IL-23/IL-17 axis, and by doing so, IL-36α amplified inflammation and the development of renal lesions (64). Besides upregulating Th17 response, IL-36 cytokines have been implicated in the induction of Th1 and Th9 responses. One study has shown that IL-36 cytokines directly induce proliferation and secretion of IL-2 in naïve CD4^+^ T cells and IL-36 cytokines act synergically with IL-12 to drive the acquisition of a Th1 phenotype characterized by the production of IFN-γ (65). Moreover, IL-36 cytokines can induce a Th9 response in T cells, which is characterized by the production of IL-9 and inhibits the proliferation of FoxP3^+^ regulatory T cells. Whereas the induction of IL-9 expression by IL-36γ was found to be dependent on IL-2/STAT5 and IL-4/STAT6 signaling, the inhibition of regulatory T cells was mediated through the impairment of the *Foxp3* locus acetylation. In conjunction, both effects contributed to colonic inflammation in an experimental oxazolone-induced colitis (66). Finally, *in vitro* analyses have determined that IL-36γ triggered IFN-γ production in CD8^+^ T cells previously stimulated with anti-CD3 (67) **(** **Figure 5B** **)**.

IL-36 cytokines exert direct effects in different myeloid cells, inducing several inflammatory processes, such as pro-inflammatory cytokine production (49, 52), DC maturation (49, 52), antimicrobial peptide production, and inflammasome activation in macrophages (64, 68). The induction of pro-inflammatory cytokines by IL-36 cytokines has been described in human monocytes and DCs, in which IL-36α, IL-36β and IL-36γ upregulated IL-6 at the transcript and protein level (49). In mice, IL-36α, IL-36β and IL-36γ induced the production of IL-6, IL-12p40, IL-12p70, CCL11, CCL4, TNF-α and G-CSF in BMDCs (52). IL-36 cytokines also induced DC maturation and increased the expression of the surface marker CD83 and the co-stimulatory molecule CD86, which are required for T cell activation (49, 52) **(** **Figure 5C** **).** In macrophages, IL-36γ induced the activation of NLRP3 inflammasome through TLR4/MyD88 pathway (64) and increased the production of the antimicrobial peptides cathelicidin and β-defensin 2 in a dose-dependent manner (68, 69). In addition, IL-36γ was shown to modulate M1 macrophage polarization (70, 71) **(** **Figure 5D** **)**. As discussed above, IL-1Rrp2 has been found to be expressed on the surface of neutrophils and B cells (50, 51). However, little is known about the effect of IL-36 cytokines on the function of these cells **(** **Figures 5E, F** **)**. Since neutrophil proteases cleave and activate IL-36 cytokines, it is speculated that neutrophils may do so to enhance their own activation and antimicrobial activity. However, this has not been elucidated to date.

## The Active Role of IL-36 Cytokines During Lung Inflammatory Diseases

The respiratory tract is anatomically compartmentalized into the upper and the lower respiratory tract (72). Both portions are susceptible to bacterial, viral, and fungal infections, although lower respiratory tract infections are associated with higher morbidity and mortality. Even though the baseline expression of IL-1Rrp2 at the transcript level is lower in lung tissue compared to that in the skin, instillation of recombinant cleaved IL-36α (73) and IL-36γ (41, 74) into the lungs of healthy mice results in rapid induction of cytokine production and neutrophil recruitment in the alveolar space, highlighting that IL-36 cytokines exert potent pro-inflammatory effects in the lungs. Numerous studies have described a major protective role for IL-36 cytokines in the host defense against pulmonary infections caused by a wide variety of pathogenic bacteria, including *Streptococcus pneumoniae* (Spn), *Klebsiella pneumoniae* (Kpn), *Legionella pneumophila* (Lpn), *Mycobacterium tuberculosis* (Mtb) and *Mycobacterium bovis* BCG (BCG) (22, 65, 68, 69, 71, 75) **(** **Table 2** **)**. In contrast with the protective effects of IL-36 cytokines on the host immune response and survival in most pulmonary bacterial infections, IL-36 cytokines have been reported to be detrimental for the host during infection with *Pseudomonas aeruginosa* (Pa) (77) **(** **Table 2** **)**. Although IL-36 signaling has also been implicated in the host immune response during influenza infection, the role of IL-36γ is controversial between studies and the apparent role of IL-36α or IL-36β has not been explored (70, 78) **(** **Table 2** **)**.

**Table 2:** Role of IL-36 cytokines during lung bacterial and viral infection and their effect in lung immunity and host survival.

Pathogen	IL-36 cytokine involved	Cellular source(s)	Effect in host survival	Effect in pathogen clearance	Effect in lung inflammation	Effect in cytokine production	Effect in immune cell recruitment/activation	References
Bacterial								
*Strepcococcus pneumoniae*	IL-36γ	Lung resident macrophage	Promotes survival	Improves bacterial clearance	Unknown	Induces	Activation of macrophages. No effect in cell recruitment.	(22, 75)
						IL-12p40 IL-23p19 IL-17 TNF-α		
						IP-10		
						IFN-γ		
*Klebsiella pneumoniae*	IL-36γ	Lung resident macrophage	Promotes survival	Improves bacterial clearance	Unknown	Induces	Unknown	(22, 75)
						IL-12		
						IL-23		
						IFN-γ		
*Legionella pneumophila*	IL-36α	Unknown	Promote survival	Improve bacterial clearance	Reduce lung injury	Unknown	Enhance neutrophil, monocyte and macrophages recruitment. Enhanced macrophage polarization and activation.	(71)
	IL-36γ							
*Mycobacterium bovis BCG*	Unknown	Unknown	None	None	Reduce lung injury	Induce	Unknown	(65, 76)
						IL-6		
						TNF-α		
						IFN-γ		
*Mycobacterium tuberculosis*	IL-36γ	Macrophage	None	Improves bacterial clearance by macrophages *in vitro*	None	None	Enhances production of antimicrobial peptides by macrophages through LXR pathway.	(68, 69, 76)
		Lung epithelial cells						
*Pseudomonas aeruginosa*	IL-36γ	Lung resident macrophage	Reduces survival	Impairment of bacterial clearance	Increase lung injury	Induces TNF-α IL-6	Impairs antimicrobial ability on macrophages through COX-2 activation and PGE2 production.	(77)
		Alveolar epithelial cells				IL-17 IL-10		

*Viral*								
*Influenza virus*	IL-36α IL-36β IL-36γ	Alveolar epithelial cells	Opposite results	Impairment of viral clearance	1.IL-36R^-/-^ mice showed reduced lung injury compared to WT mice	Induce IL-17	Enhanced neutrophil infiltration Impaired T cell activation	(70, 78)
		Neutrophils	1. L-36R^-/-^ mice improved survival. IL-36γ^-/-^ mice show equivalent survival compared to WT mice		2. IL-36γ^-/-^ mice showed severe lung injury compared to WT mice		Increased macrophage apoptosis	
			2. IL-36γ^-/-^ mice showed improved survival compared to WT mice.			CXCL-1	M1 macrophage polarization.	
						IP-10 Reduced		
						IFN-β Opposite results found in IL-6		

### Role of IL-36 Signaling During Lower Respiratory Tract Infections

The Gram-positive pathogen Spn is the most common cause of community acquired pneumonia worldwide (79) and poses a major threat to public health given its ability to cause pneumococcal invasive diseases and the emergence of multi-drug resistant clones and serotypes not covered in the current vaccines (80–82). Spn has several virulence factors on its surface that impair the action of the host complement system and antibodies, and it also possesses a polysaccharide capsule that allows it to prevent phagocytosis by macrophages and neutrophils (83, 84). During Spn infection, IL-36γ – but not IL-36α or IL-36β – is rapidly produced in the lungs by macrophages, which packages IL-36γ into microparticles and exosomes for its secretion to the extracellular space (22, 75).

The production of IL-36γ has been shown to be essential for host defense against acute pulmonary Spn infection in mice, as both genetic IL-36γ deficiency and antibody-mediated depletion of IL-36γ are associated with impaired lung bacterial clearance, increased bacterial dissemination, and reduced survival (75). Interestingly, IL-36γ deficiency did not affect neutrophil recruitment to the lungs, but instead, diminished the local production of key pro-inflammatory cytokines, including IFN-γ (75), which is well-known to activate macrophages and to enhance their ability to kill bacteria (85). Consistently, *ex vivo* stimulated macrophages derived from IL-36γ^-/-^ and IL-36R^-/-^ mice exhibited an impaired response to Spn that was characterized by a reduced phagocytic ability and reduced expression of *Nos2* (75), which encodes iNOS that is part of the killing machinery of macrophages (86). Importantly, treatment of IL-36γ^-/-^ mice with IL-36γ-loaded microparticles restored the production of IFN-γ, IL-12 and IL-23 in the lungs and improved lung Spn clearance (75). These observations indicate that IL-36γ is essential for the induction of key pro-inflammatory cytokines, including the Th1 cytokine IFN-γ, suggesting that IL-36γ is a key regulator of macrophage activation during Spn infection (75).

A similar role for IL-36γ has been described during pulmonary infection with the Gram-negative bacterium Kpn. The polysaccharide capsule of Kpn is crucial for enabling the bacteria to evade phagocytosis and escape clearance by macrophages and neutrophils (87, 88). As during Spn infection, the absence of IL-36γ during acute pulmonary Kpn infection leads to impaired bacterial clearance in the lungs, higher bacterial dissemination to the bloodstream and increased mortality. The impaired host defense in IL-36γ^-/-^ mice was characterized by impaired production of type-1 cytokines, such as IL-12, IL-23 and IFN-γ at 48 hours post infection (75).

IL-36 cytokines have also been shown to be important in host defense against infection with the Gram-negative bacterium *Legionella pneumophila* (Lpn). Lpn is the cause of Legionnaires’ disease, which can cause a severe form of pneumonia that is increasing in incidence in the United States and Europe, with an associated mortality of around 10% (89–91). A key characteristic of Lpn is the secretion of virulence factors *via* a type 4 secretion system. These secreted virulence factors prevent phagosome maturation and acidification, which allows Lpn to escape phagocytic killing by macrophages (92). In a murine model of lung Lpn infection, the expression of IL-36α and IL-36γ, but not IL-36β, is rapidly induced in the lungs following infection (71). In contrast to Spn and Kpn infection, where exclusively IL-36γ exerted the main protective effect, during Lpn infection, the combined effect of IL-36α and IL-36γ were required to protect the host and promote survival (71). The lack of IL-36 signaling was associated with reduced production of chemokines and pro-inflammatory cytokines, leading to impaired recruitment of neutrophils and monocytes into the broncho-alveolar space and to impaired lung Lpn clearance (71). In addition, ex vivo studies performed in alveolar macrophages showed that IL-36 signaling is crucial for macrophage polarization during Lpn infection, as macrophages lacking IL-1Rrp2 showed reduced expression of iNOS (M1 marker), increased expression of Arg-1 (M2 marker) and reduced production of pro-inflammatory cytokines, including IL-1β, TNF-α and IFN-γ (71).

The protective role of IL-36 cytokines has also been described in an experimental model of lung BCG infection and in Mtb infection *in vivo* and *in vitro* (65, 68, 69, 76). In the case of BCG infection, IL36R^-/-^ mice showed similar host survival (76), but resulted in excessive recruitment of immune cells and excessive lung inflammation compared to WT mice (65, 76). Moreover, isolated splenocytes from IL-36R^-/-^ mice produced lower amounts of pro-inflammatory cytokines involved in type 1 responses, such as IFN-γ, IL-6 and TNF-α after stimulation with BCG-antigen or viable bacterium *in vitro* (65). Finally, IL-36β was shown to act synergistically with IL-2 and IL-12 to promote Th1 polarization *in vitro* and to also enhance Th1 immune response *in vivo* during BCG infection (65).

Several cell types such as human peripheral blood mononuclear cells (PBMCs), human monocytes-derived macrophages, THP-1 macrophages, murine bone marrow-derived macrophages and, to a lesser extent, A549 human lung epithelial cell line, produced IL-36γ in response to live Mtb *in vitro*. This IL-36γ production was shown to depend on recognition of Mtb PAMPs *via* TLR2 and TLR4 and subsequent signaling *via* MyD88. In addition, inflammasome-dependent production of active IL-1β and IL-18 was shown to further enhance late-stage IL-36γ production in response to Mtb (68). In Mtb-infected macrophages, IL-36γ induces the activation of Liver X Receptor (LXR) (69), which actively contribute to Mtb immunity in mice by improving bacterial clearance and reducing lung inflammation (93). Mechanistically, LXR induction by IL-36γ triggered the production of antimicrobial peptides such as Cathelicidin, β-defensin 1 and β-defensin 2 (68, 69), improving Mtb clearance by macrophages. These *in vitro* findings suggest a protective role of the LXR/IL-36γ axis in response to Mtb. However, *in vivo* mouse models demonstrated that the lack of IL-36 signaling did not significantly affect host defense, lung inflammation or host survival during a pulmonary Mtb infection. This finding indicates that IL-36 signaling possess a limited contribution in the immunity against Mtb in the lungs (76).

In contrast to the beneficial effects of IL-36 cytokines in host defense against the majority of the reported bacterial infections in the lungs thus far, the effects of IL-36 cytokines are detrimental during pulmonary infection with Pa (77). Pa is a Gram-negative pathogenic bacterium that causes acute lower respiratory tract infections in the ICU (94) and chronic infections in cystic fibrosis (CF) patients. Pa pneumonia is characterized by excessive lung inflammation as a consequence of the large number of toxins and virulent factors produced and released by this pathogen to the alveolar lumen (95). Murine alveolar macrophages and alveolar epithelial cells rapidly produce high levels of IL-36α and IL-36γ within the first 24 hours following Pa infection (41, 77). The early production of IL-36γ, but not IL-36α, induces excessive production of pro-inflammatory cytokines, including TNF-α, IL-6 and IL-17 (77). IL-36γ^-/-^ and IL-36R^-/-^ mice display reduced pro-inflammatory cytokine production, reduced lung damage, and improved bacterial clearance following Pa infection compared to WT mice, suggesting that the excessive inflammation caused by IL-36γ during PA infection impairs bacterial clearance and exacerbates lung injury (77). Consistent with this finding, IL-36γ^-/-^ and IL-36R^-/-^ mice showed increased survival compared to WT mice (77). Mechanistically, IL-36γ induced the expression of COX-2 and the production of prostaglandin E2 (PGE2) in alveolar macrophages, which impaired the clearance of Pa by these cells (77). This observation shows that IL-36γ impairs bacterial clearance in a PGE2-dependent manner and is in line with previous studies that describe the immunosuppressive properties of PGE2 in the context of Pa infection (96, 97). Despite this observation, it is unclear why IL-36γ production is detrimental during acute lung Pa infection. One possibility is that Pa secretes proteases such as LasB, known to trigger endogenous host neutrophil proteases such as NE and CatG, that may result in excessive cleavage and activation of IL-36γ during Pa infection leading to uncontrolled inflammation and negatively impacting host survival (41). Further studies analyzing the role of IL-36γ examining clinical isolates with high and low proteolytic activity would provide useful insights regarding this hypothesis.

IL-36 cytokines are also important players in the lung immune response against influenza virus, although their exact function in this context remains uncertain. While one study indicates that IL-36 signaling (IL-36R^-/-^ mice) exerts detrimental effects for the host and increases mortality following infection (78), a later study indicates that IL-36γ enhances host survival (70). Using IL-36R^-/-^ mice, the first study showed that IL-36 signaling exerts a pathogenic response during pulmonary influenza virus infection, which is characterized by severe lung injury and higher mortality compared to WT mice and IL-36γ^-/-^ mice, (78). Absence of IL-36 signaling improved host survival and reduced the infiltration of neutrophils and monocytes/macrophages, reduced the production of pro-inflammatory cytokines involved in neutrophilic inflammation such as IL-6, IL-17A, CXCL-1 at day 6 post infection and limited lung inflammation and injury. Interestingly, IL-36R^-/-^ mice showed increased viral burden in the lungs, indicating that the increased resistance of IL-36R^-/-^ mice to influenza virus is determined by their ability to temper the immune response and prevent lung injury rather than due to enhanced viral clearance (78). This study did not detect a survival difference between WT and IL-36γ^-/-^ mice and suggested that the detrimental effects of IL-36 signaling in influenza virus are driven by IL-36α or IL-36β and not IL-36γ.

In contrast, another study reported that IL-36γ^-/-^ mice display a higher mortality following an acute pulmonary influenza infection and impaired viral clearance at day 6 post infection, suggesting that IL-36γ is required for an effective host defense against influenza virus (70). Interestingly, whereas no major differences in innate cell infiltration to the lungs between IL-36γ^-/-^ and WT mice were identified during the first 6 days of infection, a surprising difference was that IL-36γ^-/-^ mice showed massive apoptosis of alveolar macrophages, leading to a rapid reduction of alveolar macrophages in the lungs of these mice (70). The increased apoptosis of alveolar macrophages observed in IL-36γ^-/-^ mice was thought to be due to enhanced M2 macrophage polarization, as macrophages from IL-36γ^-/-^ mice showed higher expression of M2 markers such as CD206, CD200R in the surface, as well as *Retnla* transcript. Importantly, the transfer of WT alveolar macrophages to IL-36γ^-/-^ mice improved the survival of these mice in response to influenza to a similar level as in WT mice. Overall, these findings suggest that IL-36γ modulates alveolar macrophage polarization, thereby enhancing macrophage survival and host defense during influenza infection (70). Taken together, these two studies show that IL-36 signaling has a major role in host defense and lung inflammation during influenza infection, although the specific role of each IL-36 cytokine in host defense and lung inflammation, their differential cellular source and how their kinetic production influences the host antiviral response remains to fully understood.

### Role of IL-36 Cytokines in Non-Infectious Lung Inflammatory Diseases

The role of IL-36 cytokines in the lungs is not restricted to infectious diseases. Studies have described a potentially important role of the IL-36 pathway in asthma (98, 99), idiopathic pulmonary fibrosis (21, 100), chronic obstructive pulmonary disease (101, 102), cystic fibrosis (103) and cancer (104). During asthma, the levels of the anti-inflammatory IL36Ra in the serum and sputum of asthmatic patients are reduced compared to healthy controls (98). *In vitro* data from the same study showed that IL36Ra inhibits the production of various cytokines known to be important drivers of asthmatic inflammation, such as IL-1β, IL-6, TNF-α and IL-17A in PMBCs and in sputum mononuclear cells (SMNCs) isolated from asthmatic patients pre-stimulated with LPS, that are presumably induced by IL-36 cytokines (98). In addition, using an experimental asthma model of OVA-sensitized mice, it was established that intravenous administration of recombinant IL-36Ra (rIL-36Ra) alleviated the severity of experimental asthma by reducing airway hyper-responsiveness, alveolar inflammation and eosinophil, neutrophil, macrophage, and lymphocyte recruitment. The protective effects of rIL-36Ra were not observed in IL-36R^-/-^ mice, showing that the mechanism by which rIL-36Ra protected the lungs in sensitized mice was through the IL-36 receptor pathway (98).

The *in vivo* administration of the anti-inflammatory cytokine IL-38, which also inhibits IL-36R-signaling (55), was shown to reduce the severity of house dust mite (HDM)-induced asthma (99). Specifically, IL-38 administration decreased airway hyper-responsiveness of HDM-treated mice to baseline levels, reduced lung inflammation, BALF eosinophilic recruitment and limited the production of IL-4, IL-5, IL-6 in the lungs. Importantly, IL-38 administration also inhibited the proliferation of Th2 and Th17 cells, prevented the recruitment of ILC2 cells and promoted the proliferation of Treg cells (99). IL-36γ has been found to be upregulated during HDM-induced asthma, triggering a strong inflammatory response in the lungs of sensitized mice (74).

Idiopathic pulmonary fibrosis (IPF) is a chronic lung disease characterized by the progressive loss of lung function. Interestingly, IL-38 is highly expressed in type II pneumocytes in patients with IPF but not in healthy controls (100). Moreover, normal human lung fibroblasts express IL-1Rrp2 at baseline and produce several pro-inflammatory cytokines such as IL-8, CXCL3, G-CSF and CCL20 in response to IL-36γ (21) **(** **Figure 5G** **)**. Currently, it is not clear which factors induce IL-38 expression in IPF patients and what the role of this cytokine is in IPF progression. However, as lung fibroblasts respond to IL-36γ and IL-1Rrp2 neutralization has protective effects during intestinal fibrosis (105), it can be speculated that the IL-36 pathway may be a major pathway during lung fibrosis.

Chronic obstructive pulmonary disease (COPD) is a chronic lung inflammatory disease characterized by airflow obstruction. Promising data regarding the role of IL-36 cytokines in the development and severity of COPD has emerged in the last years. Studies have demonstrated that normal human bronchial epithelial cells and primary human bronchial epithelial cells express high amounts of IL-36α, IL-36β, IL-36γ and IL-36Ra after being treated with cigarette smoke components – a causative agent of COPD – *in vitro* (101, 102). Moreover, smokers with and without COPD presented with elevated levels of IL-36α, and IL-36γ in plasma and BALF at baseline, although no differences in IL-36 cytokines levels were found between non-COPD smokers and COPD smokers (102). Another study showed that IL-36α, and IL-36γ were increased in the sputum of neutrophilic COPD patients, compared to eosinophilic COPD, and were associated with the amount of neutrophils in the sputum (106). Despite the emergence of novel data regarding the role of IL-36 cytokines in COPD, more studies are needed to accurately understand the possible pathogenic role of IL-36 cytokines during COPD.

Cystic fibrosis (CF) is a chronic lung disorder caused by genetic mutations leading to functional defects in the cystic fibrosis transmembrane conductance regulator (CFTR), a chloride channel that regulates the flow of chloride ions in epithelial cells (107). CF is characterized by the excessive production of sticky mucus and lung inflammation caused by neutrophilic inflammation and chronic bacterial infection (107). The role of IL-36 cytokines in lung inflammation during CF remains relatively unexplored. However, CF patients usually present with a neutrophil-predominant inflammatory response in sputum supernatants, and massive release of neutrophil elastase (108), and therefore it is highly possible that IL-36 cytokines play a role in lung inflammation and injury in CF. An RNA-seq analysis of bronchial brushings obtained from CF patients and healthy controls showed that IL-36γ gene expression is upregulated in the airways of CF patients (4-fold) compared to healthy controls (103). The expression of IL-36α did not differ between CF patients and healthy controls, while the expression of IL-36β and the anti-inflammatory IL-36Ra were downregulated in CF patients (103). These data suggest that, during CF, IL-36γ but no other members of the IL-36 family may be contributing to the pro-inflammatory environment. Additional studies are needed to corroborate this hypothesis.

A potential protective role of IL-36 signaling has been identified in different types of cancer in mice and humans. In mice, IL-36γ has been shown to promote a Th1 response in a tumor model of B16 cells (67). This anti-tumor response elicited by IL-36γ was characterized by increased production of IFN-γ by CD4^+^ cells, CD8^+^ cells, NK cells and γδ T cells, which ultimately resulted in the reduction of tumor size and higher survival (67). In humans, IL-36α expression in the liver of hepatocellular carcinoma patients was found to be negatively correlated with tumor size and vascularization, while IL-36α expression was positively correlated with CD8^+^ tumor-infiltrating lymphocytes and patient survival (109). Finally, the production of IL-36γ was found to be reduced in late stages of lung squamous cell carcinoma compared to early stages (67). Although more research is needed to elucidate the exact role of IL-36 signaling during lung cancer, altogether these data suggest that IL-36 cytokines may trigger a pro-inflammatory response in the tumor microenvironment, exhibiting anti-tumoral effects in a context where pro-inflammatory signals versus immunosuppressive mechanisms determine tumor elimination or survival.

## Potential Therapeutic Modulation of IL-36 Pathway During Lung Inflammation

**Figure 6:**
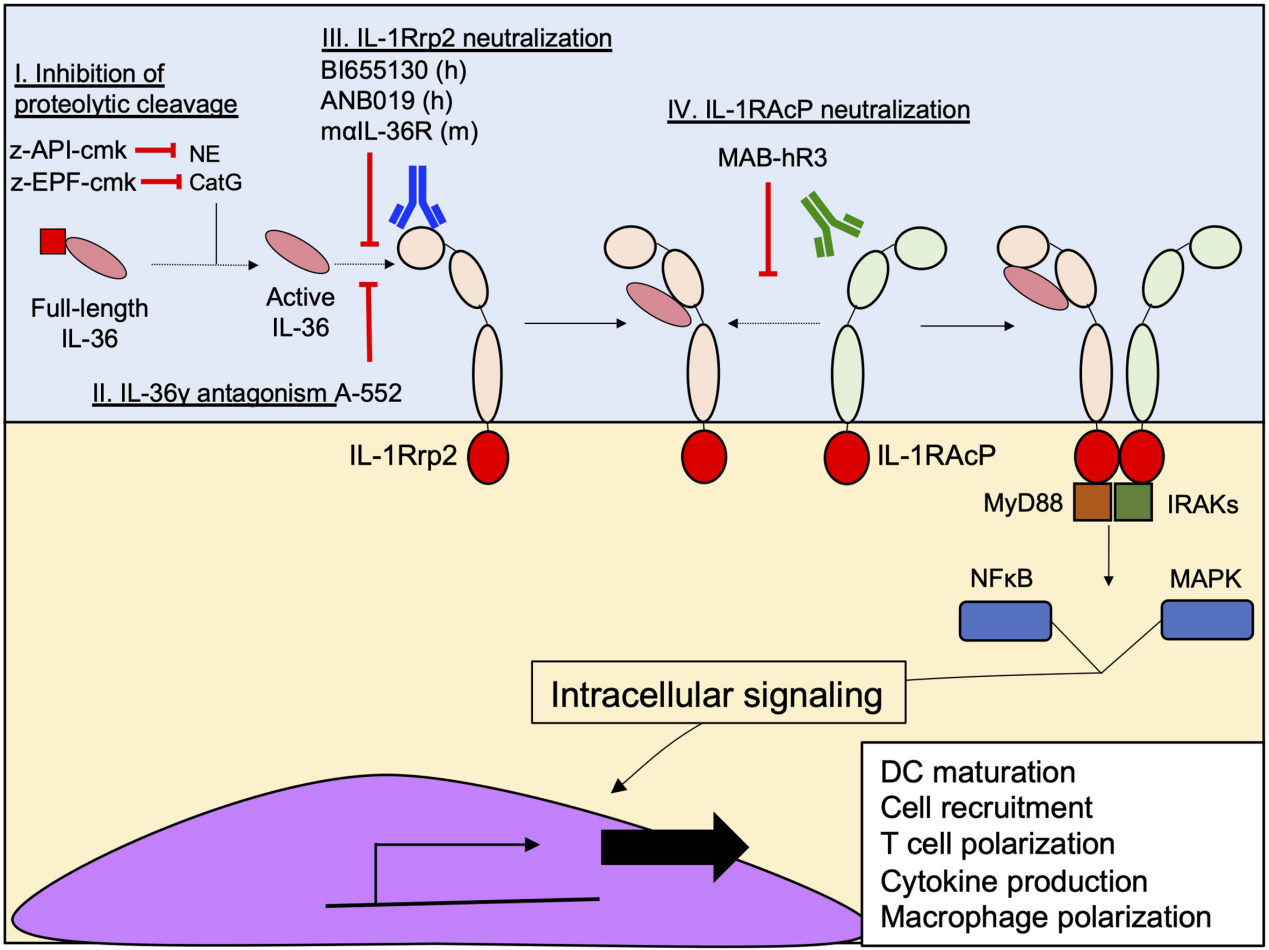
Current therapeutic strategies designed to inhibit IL-36 signaling. Several approaches have been designed to prevent the interaction between IL-36 cytokines and IL-1Rrp2, inhibiting their signaling. (I) Z-EPF-cmk and z-API-cmk Inhibit the proteolytic cleavage of IL-36 by the neutrophil proteases CatG and NE by respectively. (II) Inhibition of IL-36g-IL-1Rrp2 interaction using the small molecule A-552. (III) Monoclonal antibody-mediated neutralization of IL-1Rrp2 by human (BI655130 and ANB019) and murine (maIL-36R) antibodies; and (IV) Neutralization of IL-1RAcP using the monoclonal antibody MAB-hR3.

The initial findings regarding the pathogenic effect of IL-36 cytokines in skin psoriasis attracted the attention of scientists to therapeutically exploit the IL-36 pathway to prevent excessive inflammation **(** **Figure 6** **)** (110, 111). Indeed, a neutralizing antibody targeting IL-1Rrp2 (BI655130), designed for the treatment of generalized pustular psoriasis, was evaluated in a phase I clinical trial (NCT02978690) and showed promising results, including no adverse effects and a total pustular clearance in 6 out of 7 patients in 2 weeks of treatment (111). In addition, a second monoclonal antibody (ANB019) designed for the treatment of palmoplantar pustulosis psoriasis and generalized pustular psoriasis is currently in phase II clinical trial (NCT03633396). In addition, a monoclonal rat anti-IL-36R that recognizes the murine version of IL-1Rrp2 has been developed and characterized in mice, in terms of routes of administration, pharmacokinetics, biodistribution and excretion patterns (112). The development of this antibody opens an important window for the therapeutic potential of the IL-36 pathway in pre-clinical studies during different diseases, such as infections and non-infectious lung diseases, in which IL-36 cytokines have been identified as important effectors of inflammation.

As discussed in the previous sections, once IL-36 cytokines bind to IL-1Rrp2, a second accessory protein, IL-1RAcP is recruited and triggers downstream inflammatory pathways **(** **Figure 4A** **)**. One research group has developed a neutralizing monoclonal antibody called MAB-hR3, a humanized IgG1 Fc-LALA monoclonal antibody, – with L234A/L235A substitutions designed to prevent FcγR triggering (113) – produced in albino Zika rabbits. MAB-hR3 targets and neutralizes IL-1RAcP, blocking the IL-36γ-mediated signal *in vitro* (114). Because MAB-hR3 disrupts the recruitment of IL-1RAcP, other inflammatory signals mediated by IL-1α, IL-1β and IL-33 were also disrupted *in vitro* and *in vivo* models of local and systemic inflammation models in mice such as peritonitis, OVA-induced airway allergy and Imiquimod-induced psoriasis (114). Although the neutralization of IL-1RAcP affects the pathway induced by IL-1α, IL-1β and IL-33, its administration has positive effects in the control of the inflammation in OVA-induced allergic airway inflammation and Imiquimod-induced psoriasis. In both cases, MAB-hR3 reduced the infiltration of granulocytes and the production of pro-inflammatory cytokines (IL-6, TNF-α, IL-4, IL-5, IL-13, IL-17A, IL-17F, IL-22), reducing the intensity of the inflammation in both diseases (114).

The neutralization strategies against IL-36 signaling have not been restricted to neutralizing antibodies against IL-1Rrp2 or IL-1RAcP. The small molecule, A-552, analogue of the endothelin receptor A antagonist (Ambrisentan), has proved to be a potent antagonist of human and murine IL-36γ, but not IL-36α or IL-36β (115). The inhibitory mechanism of A-552 consists of the ability of A-552 to directly interact with the residues R^121^ and K^123^ of IL-36γ through hydrogen bonds, preventing the binding of IL-36γ to IL-1Rrp2 (115). The lack of inhibitory activity of A-552 against IL-36α or IL-36β may be explained by the inability of A-552 to establish hydrogen bonds with IL-36α and IL-36β residues (115). As previously discussed, IL-36 cytokines need to be processed in the N-terminal sequence to be fully active. This proteolytic step is critical for enhancing IL-36 activity and at least one group has taken advantage of this process through the design of peptides pseudo-substrates that specifically inhibit the proteolytic cleavage of IL-36 cytokines *in vitro* by Cathepsin G (z-EPF-cmk) and Elastase (z-API-cmk) (40). These two peptides were highly efficient in inhibiting the downstream inflammatory activity triggered by each IL-36 cytokine in transfected HeLa IL-36R^+^ cells and, in combination, completely abrogated the inflammatory effect of these cytokines (40). While targeting neutrophil proteases might seem attractive as a strategy to block IL-36 activity, targeting just neutrophil proteases may prove challenging given that other non-neutrophil proteases are capable of activating IL-36 cytokines. *In vivo* studies are needed to accurately establish the efficacy of these approaches in different inflammatory disorders.

## Conclusions and Future Perspective

IL-36 cytokines are key regulators of the host inflammatory response in infectious and non-infectious lung diseases. Several pro-inflammatory cytokines as well as various PAMPs and DAMPs trigger the expression of *il36* genes in epithelial and immune cells. When IL-36 cytokines are released into the extracellular space, these cytokines are N-terminally processed by host proteases such as cathepsin S, neutrophil elastase, cathepsin G and proteinase-3 selectively, as well as, pathogen proteases such as LasB, SpeB and Asp F13 presumably enabling more efficient binding to IL-1Rrp2. IL-1Rrp2/IL-1RAcP heterodimer downstream signaling regulates a variety of inflammatory processes in epithelial and immune cells, including cytokine production, DC maturation, T cell activation, and macrophage polarization. Whereas IL-36α and IL-36γ have been shown to be protective during lung infections with bacteria such as *K. pneumoniae*, *S. pneumoniae* and *L. pneumophilia*, IL-36 cytokines are deleterious during *P. aeruginosa* infection. Importantly, during influenza infection the role of IL-36 signaling in host defense and survival is controversial and requires further study. The role of IL-36 cytokines during coronavirus disease 2019 (COVID-19) remains to be established. Interestingly, IL36B gene expression in bronchoalveolar lavage cells of asthma patients was recently shown to correlate with bronchial epithelial cell expression of angiotensin-converting enzyme-2 (ACE2) – an enzyme that mediates the entry of severe acute respiratory syndrome coronavirus 2 (SARS-CoV-2) into host cells (116, 117).

Regarding the role of IL-36 cytokines in non-infectious lung disorders, the importance of IL-36 cytokines as a driver of inflammatory diseases such as psoriasis is well established and has generated significant interest in the development of therapeutic approaches to inhibit IL-36 signaling. The antitumoral effects of IL-36α and IL-36γ in different types of cancer further exemplify the importance of the IL-36 pathway in enhancing host immune responses, while the strong ability of IL-36γ to induce lung inflammation in the context of asthma, as well as in COPD, emphasizes that the IL-36 pathway needs to be tightly regulated to avoid excessive pathological inflammatory responses. Although IL-36 cytokines have been shown to be upregulated in the lungs of CF patients, further research is needed to establish whether IL-36 signaling plays a protective or deleterious role in the context of CF.

The differential roles of IL-36α, IL-36β and IL-36γ in models of lung inflammation suggest a degree of specificity regarding the responses mediated by the individual IL-36 cytokine in the lungs. How such specificity is achieved is currently not known. Although the recognition of each IL-36 cytokine by IL-1Rrp2 is unique in terms of affinity and specific aminoacidic interactions (54, 56), all the IL-36 family members – except for the antagonistic IL-36Ra – appear to activate similar intracellular pathways that involve MYD88/IRAK and MAPK/NF-kB signaling. Moreover, a recent study showed that whereas isolated murine macrophages and fibroblasts express high levels of IL-36γ in response to heat-killed Kpn, type II alveolar epithelial cells expressed higher levels of IL-36α compared to IL-36γ (102). Finally, given that there is significant overlap in the proteases by which each IL-36 cytokines are activated, we speculate that the distinct roles for individual IL-36 cytokines are influenced by the local milieu. Considering this, we previously reported that *Pa* protease drives the activity of endogenous host proteases such as NE (47). In addition, LasB cleaves IL-36γ proximal to M^19^, and *in silico* docking analyses predict that the M^19^ and bioactive S^18^ isoforms bind IL-1Rrp2 similarly (41). Also, specific proteases from *S. pyogenes* (SpeB) and *Aspergillus fumigatus* (Asp F13) process IL-36γ to generate the bioactive isoform S^18^ *in vitro* (45). Finally, unidentified proteases from *S. aureus* and *T. rubrum* were also found to generate the S^18^ isoform of IL-36γ *in vitro* (45). Therefore, distinct pathogens generate a local inflammatory and proteolytic environment favoring the induction of and processing of IL-36γ over IL-36α and IL-36β.

Since the discovery of IL-36 cytokines two decades ago, research has provided extensive information about IL-36 cytokine biology and their role in inflammation, putting IL-36 cytokines forward as central effectors of lung inflammation. The IL-36 pathway may be a promising target for therapeutic strategies that focus on modulating the host inflammatory response during inflammatory disorders in the lungs. Therefore, there is a clear need to continue to elucidate the complex regulation of IL-36 cytokines, and biology of IL-36 cytokines in infectious and non-infectious lung diseases.

## Author Contributions

All authors listed have made a substantial, direct, and intellectual contribution to the work, and approved it for publication.

## Funding

This work was supported in part by the University of Pittsburgh Vascular Medicine Institute, the Hemophilia Center of Western Pennsylvania, and the Institute for Transfusion Medicine (HP), the National Heart, Lung, And Blood Institute of the National Institutes of Health under Award Numbers; P01 HL114453, R01 HL136143, R01 HL142084, K24 HL143285 (JL), R01 HL123515 (TS).

## Author Disclaimer

The content is solely the responsibility of the authors and does not necessarily represent the official views of the National Institutes of Health or any other sponsoring agency.

## Conflict of Interest

JL discloses a paid consultantship with Janssen Pharmaceuticals, Inc. that is unrelated to the work presented in this manuscript.

The remaining authors declare that the research was conducted in the absence of any commercial or financial relationships that could be construed as a potential conflict of interest.

## Publisher’s Note

All claims expressed in this article are solely those of the authors and do not necessarily represent those of their affiliated organizations, or those of the publisher, the editors and the reviewers. Any product that may be evaluated in this article, or claim that may be made by its manufacturer, is not guaranteed or endorsed by the publisher.

## Glossary

**Table:**

AbR	Aryl hydrocarbon receptor
ACE-2	Angiotensin-converting enzyme-2
ARE	Antioxidant Response Elements
Arg-1	Arginase-1
BALF	Broncho-alveolar lavage fluid
BCG	*Mycobacterium bovis* BCG
BMDCs	bone-marrow-derived dendritic cells
BMDMs	Bone marrow derived macrophages
CatG	Cathepsin G
CatS	Cathepsin S
CCL4	C-C Motif Chemokine Ligand 4
CCL11	C-C Motif Chemokine Ligand 11
CCL20	C-C Motif Chemokine Ligand 20
CF	Cystic fibrosis
CFTR	Cystic fibrosis transmembrane conductance regulator
COPD	Chronic obstructive pulmonary disease
COVID-19	Coronavirus disease 2019
COX-2	Cyclooxygenase-2
CXCL3	C-X-C Motif Chemokine Ligand 3
CXCL-8	C-X-C Motif Chemokine Ligand 8
DCs	dendritic cells
dsRNA	Double strain RNA
DSS	Dextran Sulfate Sodium
ERK1/2	Extracellular signal-regulated kinase &frac12;
F13	Aspergillus factor 13
FcγR	Fc gamma receptors
FoxP3	Forkhead box P3
FSL-1	Trifluoroacetate salt
GM-CSF	Granulocyte-macrophage colony-stimulating factor
G-CSF	Granulocyte-colony stimulating factor
HDM	House dust mite
IκBζ	NF-κB inhibitor zeta
ICU	Intensive care unit
IFN-γ	Interferon gamma
iNOS	Inducible nitric oxide synthase
IL-1F5	Interleukin-1 family member 5
IL-1F6	Interleukin-1 family member 6
IL-1F8	Interleukin-1 family member 8
IL-1F9	Interleukin-1 family member 9
IL-1α	Interleukin-1 alpha
IL-1β	Interleukin-1 beta
IL-1Rrp2	IL-1 Receptor-Related protein 2
IL-1RAcP	Interleukin-1 receptor accessory protein
IL-2	Interleukin-2
IL-4	Interleukin-4
IL-5	Interleukin-5
IL-6	Interleukin-6
IL-8	Interleukin-8
IL-9	Interleukin-9
IL-12	Interleukin-12
IL-13	Interleukin-13
IL-17A	Interleukin-17A
IL-17F	Interleukin-17F
IL-18	Interleukin-18
IL-22	Interleukin-22
IL-23A	Interleukin-23A
IL-33	Interleukin-33
IL-36α	Interleukin-36 alpha
IL-36β	Interleukin-36 beta
IL-36γ	Interleukin-36 gamma
IL-36Ra	Interleukin-36 receptor antagonist
ILC2	Type 2 innate lymphoid cells
IPF	Idiopathic pulmonary fibrosis
IRAK	Interleukin-1 receptor-associated kinase
IRF6	Interferon response factor 6
JNK1/2	c-Jun NH2-terminal kinase 1/2
Kpn	*Klebsiella pneumoniae*
LasB	Elastase B
Lpn	*Legionella pneumophila*
LPS	Lipopolysaccharides
LXR	Liver X Receptor
MAPK	Mitogen-activated protein kinase
MIKs	immortalized keratinocytes
Mtb	*Mycobacterium tuberculosis*
MyD88	Myeloid differentiation primary response 88
NE	Neutrophil elastase
NF-κB	Nuclear Factor kappa-light-chain-enhancer of activated B cells
Notch2	Neurogenic locus notch homolog protein 2
Nrf2	Nuclear factor erythroid 2
OVA	Ovalbumin
P3	Proteinase-3
P-TEFb	Positive transcription elongation factor
Pa	*Pseudomonas aeruginosa*
PAM_3_CSK_4_	N-Palmitoyl-S-[2,3-bis(palmitoyloxy)-(2RS)-propyl]- [R]-cysteinyl-[S]-seryl-[S]-lysyl-[S]-lysyl-[S]-lysyl-[S]-lysine
PAMPs	Pathogen-associated molecular patterns
PBMCs	Peripheral blood mononuclear cells
PGE2	Prostaglandin E2
PMA	Phorbol 12-myristate 13-acetate
Poly I:C	Polyinosinic:polycytidylic acid
*Retnla*	Resistin-like alpha transcript
SARS-CoV-2	Severe acute respiratory syndrome coronavirus 2
SEC	Super elongation complex
SMNCs	Sputum mononuclear cells
SpeB	Streptococcal pyrogenic exotoxin B
Spn	*Streptococcus pneumoniae*
STAT	Signal transducer and activator of transcription
T-bet	T-box transcription factor TBX21
tBHQ	tert-butylhydroquinone
TIR	Toll/interleukin-1 receptor
Th1	T helper cell type 1
Th2	T helper cell type 2
Th9	T helper cell type 9
Th17	T helper cell type 17
TLR	Toll-like receptor
TNF-α	Tumour necrosis factor alpha
TRIF	TIR-domain-containing adapter-inducing interferon-β
VDR	Vitamin D receptor

## References

[1] Bassoy EY Towne JE Gabay C. Regulation and Function of Interleukin-36 Cytokines. Immunol Rev (2018) 281(1):169–78. doi: 10.1111/imr.12610 29247994

[2] Dunn E Sims JE Nicklin MJ O’Neill LA. Annotating Genes With Potential Roles in the Immune System: Six New Members of the IL-1 Family. Trends Immunol (2001) 22(10):533–6. doi: 10.1016/s1471-4906(01)02034-8 11574261

[3] Nicklin MJ Barton JL Nguyen M FitzGerald MG Duff GW Kornman K. A Sequence-Based Map of the Nine Genes of the Human Interleukin-1 Cluster. Genomics (2002) 79(5):718–25. doi: 10.1006/geno.2002.6751 11991722

[4] Taylor SL Renshaw BR Garka KE Smith DE Sims JE. Genomic Organization of the Interleukin-1 Locus. Genomics (2002) 79(5):726–33. doi: 10.1006/geno.2002.6752 11991723

[5] Kumar S McDonnell PC Lehr R Tierney L Tzimas MN Griswold DE. Identification and Initial Characterization of Four Novel Members of the Interleukin-1 Family. J Biol Chem (2000) 275(14):10308–14. doi: 10.1074/jbc.275.14.10308 10744718

[6] Busfield SJ Comrack CA Yu G Chickering TW Smutko JS Zhou H. Identification and Gene Organization of Three Novel Members of the IL-1 Family on Human Chromosome 2. Genomics (2000) 66(2):213–6. doi: 10.1006/geno.2000.6184 10860666

[7] Smith DE Renshaw BR Ketchem RR Kubin M Garka KE Sims JE. Four New Members Expand the Interleukin-1 Superfamily. J Biol Chem (2000) 275(2):1169–75. doi: 10.1074/jbc.275.2.1169 10625660

[8] Debets R Timans JC Homey B Zurawski S Sana TR Lo S. Two Novel IL-1 Family Members, IL-1 Delta and IL-1 Epsilon, Function as an Antagonist and Agonist of NF-Kappa B Activation Through the Orphan IL-1 Receptor-Related Protein 2. J Immunol (2001) 167(3):1440–6. doi: 10.4049/jimmunol.167.3.1440 11466363

[9] Dinarello C Arend W Sims J Smith D Blumberg H O’Neill L. IL-1 Family Nomenclature. Nat Immunol (2010) 11(11):973. doi: 10.1038/ni1110-973 20959797 PMC4174560

[10] Sims JE Nicklin MJ Bazan JF Barton JL Busfield SJ Ford JE. A New Nomenclature for IL-1-Family Genes. Trends Immunol (2001) 22(10):536–7. doi: 10.1016/s1471-4906(01)02040-3 11574262

[11] Towne JE Garka KE Renshaw BR Virca GD Sims JE. Interleukin (IL)-1f6, IL-1F8, and IL-1f9 Signal Through IL-1Rrp2 and IL-1racp to Activate the Pathway Leading to NF-kappaB and MAPKs. J Biol Chem (2004) 279(14):13677–88. doi: 10.1074/jbc.M400117200 14734551

[12] Wesche H Korherr C Kracht M Falk W Resch K Martin MU. The Interleukin-1 Receptor Accessory Protein (IL-1racp) Is Essential for IL-1-Induced Activation of Interleukin-1 Receptor-Associated Kinase (IRAK) and Stress-Activated Protein Kinases (SAP Kinases). J Biol Chem (1997) 272(12):7727–31. doi: 10.1074/jbc.272.12.7727 9065432

[13] Arend WP. Interleukin-1 Receptor Antagonist. Adv Immunol (1993) 54:167–227. doi: 10.1016/s0065-2776(08)60535-0 8379462

[14] Towne JE Renshaw BR Douangpanya J Lipsky BP Shen M Gabel CA. Interleukin-36 (IL-36) Ligands Require Processing for Full Agonist (IL-36alpha, IL-36beta, and IL-36gamma) or Antagonist (IL-36ra) Activity. J Biol Chem (2011) 286(49):42594–602. doi: 10.1074/jbc.M111.267922 PMC3234937 21965679

[15] Buhl AL Wenzel J. Interleukin-36 in Infectious and Inflammatory Skin Diseases. Front Immunol (2019) 10:1162. doi: 10.3389/fimmu.2019.01162 31191535 PMC6545975

[16] Fagerberg L Hallstrom BM Oksvold P Kampf C Djureinovic D Odeberg J. Analysis of the Human Tissue-Specific Expression by Genome-Wide Integration of Transcriptomics and Antibody-Based Proteomics. Mol Cell Proteomics (2014) 13(2):397–406. doi: 10.1074/mcp.M113.035600 24309898 PMC3916642

[17] Jiang Z Liu Y Li C Chang L Wang W Wang Z. IL-36gamma Induced by the TLR3-SLUG-VDR Axis Promotes Wound Healing *via* REG3A. J Invest Dermatol (2017) 137(12):2620–9. doi: 10.1016/j.jid.2017.07.820 28774595

[18] Tortola L Rosenwald E Abel B Blumberg H Schafer M Coyle AJ. Psoriasiform Dermatitis is Driven by IL-36-Mediated DC-Keratinocyte Crosstalk. J Clin Invest (2012) 122(11):3965–76. doi: 10.1172/JCI63451 PMC3484446 23064362

[19] Winkle SM Throop AL Herbst-Kralovetz MM. IL-36gamma Augments Host Defense and Immune Responses in Human Female Reproductive Tract Epithelial Cells. Front Microbiol (2016) 7:955. doi: 10.3389/fmicb.2016.00955 27379082 PMC4911402

[20] Huynh J Scholz GM Aw J Kwa MQ Achuthan A Hamilton JA. IRF6 Regulates the Expression of IL-36gamma by Human Oral Epithelial Cells in Response to Porphyromonas Gingivalis. J Immunol (2016) 196(5):2230–8. doi: 10.4049/jimmunol.1501263 26819203

[21] Chustz RT Nagarkar DR Poposki JA Favoreto S Jr. Avila PC Schleimer RP. Regulation and Function of the IL-1 Family Cytokine IL-1F9 in Human Bronchial Epithelial Cells. Am J Respir Cell Mol Biol (2011) 45(1):145–53. doi: 10.1165/rcmb.2010-0075OC PMC3145067 20870894

[22] Kovach MA Singer BH Newstead MW Zeng X Moore TA White ES. IL-36gamma is Secreted in Microparticles and Exosomes by Lung Macrophages in Response to Bacteria and Bacterial Components. J Leukoc Biol (2016) 100(2):413–21. doi: 10.1189/jlb.4A0315-087R PMC4945350 26864267

[23] Verma AH Zafar H Ponde NO Hepworth OW Sihra D Aggor FEY. IL-36 and IL-1/IL-17 Drive Immunity to Oral Candidiasis via Parallel Mechanisms. J Immunol (2018) 201(2):627–34. doi: 10.4049/jimmunol.1800515 PMC6039262 29891557

[24] Taghavi M Khosravi A Mortaz E Nikaein D Athari SS. Role of Pathogen-Associated Molecular Patterns (PAMPS) in Immune Responses to Fungal Infections. Eur J Pharmacol (2017) 808:8–13. doi: 10.1016/j.ejphar.2016.11.013 27851904

[25] Takahashi K Nishida A Shioya M Imaeda H Bamba S Inatomi O. Interleukin (IL)-1beta Is a Strong Inducer of IL-36gamma Expression in Human Colonic Myofibroblasts. PloS One (2015) 10(11):e0138423. doi: 10.1371/journal.pone.0138423 26562662 PMC4643060

[26] Bachmann M Scheiermann P Hardle L Pfeilschifter J Muhl H. IL-36gamma/IL-1F9, an Innate T-Bet Target in Myeloid Cells. J Biol Chem (2012) 287(50):41684–96. doi: 10.1074/jbc.M112.385443 PMC3516718 23095752

[27] Wajant H Pfizenmaier K Scheurich P. Tumor Necrosis Factor Signaling. Cell Death Differ (2003) 10(1):45–65. doi: 10.1038/sj.cdd.4401189 12655295

[28] Eyerich S Eyerich K Cavani A Schmidt-Weber C. IL-17 and IL-22: Siblings, Not Twins. Trends Immunol (2010) 31(9):354–61. doi: 10.1016/j.it.2010.06.004 20691634

[29] Jensen LE. Interleukin-36 Cytokines may Overcome Microbial Immune Evasion Strategies That Inhibit Interleukin-1 Family Signaling. Sci Signal (2017) 10(492). doi: 10.1126/scisignal.aan3589 28811383

[30] Palomo J Dietrich D Martin P Palmer G Gabay C. The Interleukin (IL)-1 Cytokine Family–Balance Between Agonists and Antagonists in Inflammatory Diseases. Cytokine (2015) 76(1):25–37. doi: 10.1016/j.cyto.2015.06.017 26185894

[31] Bhat MY Solanki HS Advani J Khan AA Keshava Prasad TS Gowda H. Comprehensive Network Map of Interferon Gamma Signaling. J Cell Commun Signal (2018) 12(4):745–51. doi: 10.1007/s12079-018-0486-y PMC6235777 30191398

[32] Lighvani AA Frucht DM Jankovic D Yamane H Aliberti J Hissong BD. T-Bet is Rapidly Induced by Interferon-Gamma in Lymphoid and Myeloid Cells. Proc Natl Acad Sci USA (2001) 98(26):15137–42. doi: 10.1073/pnas.261570598 PMC64996 11752460

[33] Zimmermann J Kuhl AA Weber M Grun JR Loffler J Haftmann C. T-Bet Expression by Th Cells Promotes Type 1 Inflammation But Is Dispensable for Colitis. Mucosal Immunol (2016) 9(6):1487–99. doi: 10.1038/mi.2016.5 26883725

[34] Hertweck A Evans CM Eskandarpour M Lau JC Oleinika K Jackson I. T-Bet Activates Th1 Genes Through Mediator and the Super Elongation Complex. Cell Rep (2016) 15(12):2756–70. doi: 10.1016/j.celrep.2016.05.054 PMC4920892 27292648

[35] Kurinna S Muzumdar S Kohler UA Kockmann T Auf dem Keller U Schafer M. Autocrine and Paracrine Regulation of Keratinocyte Proliferation Through a Novel Nrf2-IL-36gamma Pathway. J Immunol (2016) 196(11):4663–70. doi: 10.4049/jimmunol.1501447 27183581

[36] Henry CM Sullivan GP Clancy DM Afonina IS Kulms D Martin SJ. Neutrophil-Derived Proteases Escalate Inflammation Through Activation of IL-36 Family Cytokines. Cell Rep (2016) 14(4):708–22. doi: 10.1016/j.celrep.2015.12.072 26776523

[37] Clancy DM Sullivan GP Moran HBT Henry CM Reeves EP McElvaney NG. Extracellular Neutrophil Proteases Are Efficient Regulators of IL-1, IL-33, and IL-36 Cytokine Activity But Poor Effectors of Microbial Killing. Cell Rep (2018) 22(11):2937–50. doi: 10.1016/j.celrep.2018.02.062 29539422

[38] Macleod T Doble R McGonagle D Wasson CW Alase A Stacey M. Neutrophil Elastase-Mediated Proteolysis Activates the Anti-Inflammatory Cytokine IL-36 Receptor Antagonist. Sci Rep (2016) 6:24880. doi: 10.1038/srep24880 27101808 PMC4840362

[39] Ainscough JS Macleod T McGonagle D Brakefield R Baron JM Alase A. Cathepsin S Is the Major Activator of the Psoriasis-Associated Proinflammatory Cytokine IL-36gamma. Proc Natl Acad Sci USA (2017) 114(13):E2748–57. doi: 10.1073/pnas.1620954114 PMC5380102 28289191

[40] Sullivan GP Henry CM Clancy DM Mametnabiev T Belotcerkovskaya E Davidovich P. Suppressing IL-36-Driven Inflammation Using Peptide Pseudosubstrates for Neutrophil Proteases. Cell Death Dis (2018) 9(3):378. doi: 10.1038/s41419-018-0385-4 29515113 PMC5841435

[41] Peñaloza HF Olonisakin TF Bain WG Qu Y van der Geest R Zupetic J. Thrombospondin-1 Restricts Interleukin-36gamma-Mediated Neutrophilic Inflammation During Pseudomonas Aeruginosa Pulmonary Infection. mBio (2021) 12(2). doi: 10.1128/mBio.03336-20 PMC8092289 33824208

[42] Small DM Brown RR Doherty DF Abladey A Zhou-Suckow Z Delaney RJ. Targeting of Cathepsin S Reduces Cystic Fibrosis-Like Lung Disease. Eur Respir J (2019) 53(3). doi: 10.1183/13993003.01523-2018 30655278

[43] Beers C Burich A Kleijmeer MJ Griffith JM Wong P Rudensky AY. Cathepsin S Controls MHC Class II-Mediated Antigen Presentation by Epithelial Cells *In Vivo* . J Immunol (2005) 174(3):1205–12. doi: 10.4049/jimmunol.174.3.1205 15661874

[44] Wartenberg M Saidi A Galibert M Joulin-Giet A Burlaud-Gaillard J Lecaille F. Imaging of Extracellular Cathepsin S Activity by a Selective Near Infrared Fluorescence Substrate-Based Probe. Biochimie (2019) 166:84–93. doi: 10.1016/j.biochi.2019.03.013 30914255

[45] Macleod T Ainscough JS Hesse C Konzok S Braun A Buhl AL. The Proinflammatory Cytokine IL-36gamma Is a Global Discriminator of Harmless Microbes and Invasive Pathogens Within Epithelial Tissues. Cell Rep (2020) 33(11):108515. doi: 10.1016/j.celrep.2020.108515 33326792 PMC7758160

[46] Zhao Y Olonisakin TF Xiong Z Hulver M Sayeed S Yu MT. Thrombospondin-1 Restrains Neutrophil Granule Serine Protease Function and Regulates the Innate Immune Response During Klebsiella Pneumoniae Infection. Mucosal Immunol (2015) 8(4):896–905. doi: 10.1038/mi.2014.120 25492474 PMC4465063

[47] Qu Y Olonisakin T Bain W Zupetic J Brown R Hulver M. Thrombospondin-1 Protects Against Pathogen-Induced Lung Injury by Limiting Extracellular Matrix Proteolysis. JCI Insight (2018) 3(3). doi: 10.1172/jci.insight.96914 PMC5821195 29415890

[48] Madonna S Girolomoni G Dinarello CA Albanesi C. The Significance of IL-36 Hyperactivation and IL-36r Targeting in Psoriasis. Int J Mol Sci 20(13):3318. doi: 10.3390/ijms20133318 PMC6650959 31284527

[49] Foster AM Baliwag J Chen CS Guzman AM Stoll SW Gudjonsson JE. IL-36 Promotes Myeloid Cell Infiltration, Activation, and Inflammatory Activity in Skin. J Immunol (2014) 192(12):6053–61. doi: 10.4049/jimmunol.1301481 PMC4048788 24829417

[50] Penha R Higgins J Mutamba S Barrow P Mahida Y Foster N. IL-36 Receptor Is Expressed by Human Blood and Intestinal T Lymphocytes and is Dose-Dependently Activated *via* IL-36beta and Induces CD4+ Lymphocyte Proliferation. Cytokine (2016) 85:18–25. doi: 10.1016/j.cyto.2016.05.023 27269181

[51] Wang H Li ZY Jiang WX Liao B Zhai GT Wang N. The Activation and Function of IL-36gamma in Neutrophilic Inflammation in Chronic Rhinosinusitis. J Allergy Clin Immunol (2018) 141(5):1646–58. doi: 10.1016/j.jaci.2017.12.972 29274415

[52] Vigne S Palmer G Lamacchia C Martin P Talabot-Ayer D Rodriguez E. IL-36r Ligands Are Potent Regulators of Dendritic and T Cells. Blood (2011) 118(22):5813–23. doi: 10.1182/blood-2011-05-356873 21860022

[53] Greenfeder SA Nunes P Kwee L Labow M Chizzonite RA Ju G. Molecular Cloning and Characterization of a Second Subunit of the Interleukin 1 Receptor Complex. J Biol Chem (1995) 270(23):13757–65. doi: 10.1074/jbc.270.23.13757 7775431

[54] Zhou L Todorovic V Kakavas S Sielaff B Medina L Wang L. Quantitative Ligand and Receptor Binding Studies Reveal the Mechanism of Interleukin-36 (IL-36) Pathway Activation. J Biol Chem (2018) 293(2):403–11. doi: 10.1074/jbc.M117.805739 PMC5767850 29180446

[55] van de Veerdonk FL Stoeckman AK Wu G Boeckermann AN Azam T Netea MG. IL-38 Binds to the IL-36 Receptor and has Biological Effects on Immune Cells Similar to IL-36 Receptor Antagonist. Proc Natl Acad Sci USA (2012) 109(8):3001–5. doi: 10.1073/pnas.1121534109 PMC3286950 22315422

[56] Yi G Ybe JA Saha SS Caviness G Raymond E Ganesan R. Structural and Functional Attributes of the Interleukin-36 Receptor. J Biol Chem (2016) 291(32):16597–609. doi: 10.1074/jbc.M116.723064 PMC4974375 27307043

[57] Gunther S Sundberg EJ. Molecular Determinants of Agonist and Antagonist Signaling Through the IL-36 Receptor. J Immunol (2014) 193(2):921–30. doi: 10.4049/jimmunol.1400538 24935927

[58] Dietrich D Gabay C. Inflammation: IL-36 has Proinflammatory Effects in Skin But Not in Joints. Nat Rev Rheumatol (2014) 10(11):639–40. doi: 10.1038/nrrheum.2014.156 25201385

[59] Swindell WR Beamer MA Sarkar MK Loftus S Fullmer J Xing X. RNA-Seq Analysis of IL-1B and IL-36 Responses in Epidermal Keratinocytes Identifies a Shared MyD88-Dependent Gene Signature. Front Immunol (2018) 9:80. doi: 10.3389/fimmu.2018.00080 29434599 PMC5796909

[60] Muller A Hennig A Lorscheid S Grondona P Schulze-Osthoff K Hailfinger S. IkappaBzeta Is a Key Transcriptional Regulator of IL-36-Driven Psoriasis-Related Gene Expression in Keratinocytes. Proc Natl Acad Sci USA (2018) 115(40):10088–93. doi: 10.1073/pnas.1801377115 PMC6176600 30224457

[61] Carrier Y Ma HL Ramon HE Napierata L Small C O’Toole M. Inter-Regulation of Th17 Cytokines and the IL-36 Cytokines *In Vitro* and *In Vivo*: Implications in Psoriasis Pathogenesis. J Invest Dermatol (2011) 131(12):2428–37. doi: 10.1038/jid.2011.234 21881584

[62] Ngo VL Abo H Maxim E Harusato A Geem D Medina-Contreras O. A Cytokine Network Involving IL-36gamma, IL-23, and IL-22 Promotes Antimicrobial Defense and Recovery From Intestinal Barrier Damage. Proc Natl Acad Sci USA (2018) 115(22):E5076–85. doi: 10.1073/pnas.1718902115 PMC5984499 29760082

[63] Ngo VL Abo H Kuczma M Szurek E Moore N Medina-Contreras O. IL-36r Signaling Integrates Innate and Adaptive Immune-Mediated Protection Against Enteropathogenic Bacteria. Proc Natl Acad Sci USA (2020) 117(44):27540–8. doi: 10.1073/pnas.2004484117 PMC7959549 33087566

[64] Chi HH Hua KF Lin YC Chu CL Hsieh CY Hsu YJ. IL-36 Signaling Facilitates Activation of the NLRP3 Inflammasome and IL-23/IL-17 Axis in Renal Inflammation and Fibrosis. J Am Soc Nephrol (2017) 28(7):2022–37. doi: 10.1681/ASN.2016080840 PMC5491282 28179433

[65] Vigne S Palmer G Martin P Lamacchia C Strebel D Rodriguez E. IL-36 Signaling Amplifies Th1 Responses by Enhancing Proliferation and Th1 Polarization of Naive CD4+ T Cells. Blood (2012) 120(17):3478–87. doi: 10.1182/blood-2012-06-439026 22968459

[66] Harusato A Abo H Ngo VL Yi SW Mitsutake K Osuka S. IL-36gamma Signaling Controls the Induced Regulatory T Cell-Th9 Cell Balance *via* NFkappaB Activation and STAT Transcription Factors. Mucosal Immunol (2017) 10(6):1455–67. doi: 10.1038/mi.2017.21 PMC5610052 28327619

[67] Wang X Zhao X Feng C Weinstein A Xia R Wen W. IL-36gamma Transforms the Tumor Microenvironment and Promotes Type 1 Lymphocyte-Mediated Antitumor Immune Responses. Cancer Cell (2015) 28(3):296–306. doi: 10.1016/j.ccell.2015.07.014 26321222 PMC4573903

[68] Ahsan F Moura-Alves P Guhlich-Bornhof U Klemm M Kaufmann SH Maertzdorf J. Role of Interleukin 36gamma in Host Defense Against Tuberculosis. J Infect Dis (2016) 214(3):464–74. doi: 10.1093/infdis/jiw152 27389350

[69] Ahsan F Maertzdorf J Guhlich-Bornhof U Kaufmann SHE Moura-Alves P. IL-36/LXR Axis Modulates Cholesterol Metabolism and Immune Defense to Mycobacterium Tuberculosis. Sci Rep (2018) 8(1):1520. doi: 10.1038/s41598-018-19476-x 29367626 PMC5784124

[70] Wein AN Dunbar PR McMaster SR Li ZT Denning TL Kohlmeier JE. IL-36gamma Protects Against Severe Influenza Infection by Promoting Lung Alveolar Macrophage Survival and Limiting Viral Replication. J Immunol (2018) 201(2):573–82. doi: 10.4049/jimmunol.1701796 PMC6089355 29848754

[71] Nanjo Y Newstead MW Aoyagi T Zeng X Takahashi K Yu FS. Overlapping Roles for Interleukin-36 Cytokines in Protective Host Defense Against Murine Legionella Pneumophila Pneumonia. Infect Immun (2019) 87(1). doi: 10.1128/IAI.00583-18 PMC6300640 30323031

[72] Man WH de Steenhuijsen Piters WA Bogaert D. The Microbiota of the Respiratory Tract: Gatekeeper to Respiratory Health. Nat Rev Microbiol (2017) 15(5):259–70. doi: 10.1038/nrmicro.2017.14 PMC7097736 28316330

[73] Ramadas RA Ewart SL Iwakura Y Medoff BD LeVine AM. IL-36alpha Exerts Pro-Inflammatory Effects in the Lungs of Mice. PloS One (2012) 7(9):e45784. doi: 10.1371/journal.pone.0045784 23029241 PMC3447790

[74] Ramadas RA Ewart SL Medoff BD LeVine AM. Interleukin-1 Family Member 9 Stimulates Chemokine Production and Neutrophil Influx in Mouse Lungs. Am J Respir Cell Mol Biol (2011) 44(2):134–45. doi: 10.1165/rcmb.2009-0315OC PMC3049228 20299540

[75] Kovach MA Singer B Martinez-Colon G Newstead MW Zeng X Mancuso P. IL-36gamma is a Crucial Proximal Component of Protective Type-1-Mediated Lung Mucosal Immunity in Gram-Positive and -Negative Bacterial Pneumonia. Mucosal Immunol (2017) 10(5):1320–34. doi: 10.1038/mi.2016.130 PMC5548659 28176791

[76] Segueni N Vigne S Palmer G Bourigault ML Olleros ML Vesin D. Limited Contribution of IL-36 Versus IL-1 and TNF Pathways in Host Response to Mycobacterial Infection. PloS One (2015) 10(5):e0126058. doi: 10.1371/journal.pone.0126058 25950182 PMC4423901

[77] Aoyagi T Newstead MW Zeng X Nanjo Y Peters-Golden M Kaku M. Interleukin-36gamma and IL-36 Receptor Signaling Mediate Impaired Host Immunity and Lung Injury in Cytotoxic Pseudomonas Aeruginosa Pulmonary Infection: Role of Prostaglandin E2. PloS Pathog (2017) 13(11):e1006737. doi: 10.1371/journal.ppat.1006737 29166668 PMC5718565

[78] Aoyagi T Newstead MW Zeng X Kunkel SL Kaku M Standiford TJ. IL-36 Receptor Deletion Attenuates Lung Injury and Decreases Mortality in Murine Influenza Pneumonia. Mucosal Immunol (2017) 10(4):1043–55. doi: 10.1038/mi.2016.107 PMC5471142 27966554

[79] Garau J Calbo E. Community-Acquired Pneumonia. Lancet (2008) 371(9611):455–8. doi: 10.1016/S0140-6736(08)60216-0 18262027

[80] Cherazard R Epstein M Doan TL Salim T Bharti S Smith MA. Antimicrobial Resistant Streptococcus Pneumoniae: Prevalence, Mechanisms, and Clinical Implications. Am J Ther (2017) 24(3):e361–9. doi: 10.1097/MJT.0000000000000551 28430673

[81] Choe YJ Lee HJ Lee H Oh CE Cho EY Choi JH. Emergence of Antibiotic-Resistant non-Vaccine Serotype Pneumococci in Nasopharyngeal Carriage in Children After the Use of Extended-Valency Pneumococcal Conjugate Vaccines in Korea. Vaccine (2016) 34(40):4771–6. doi: 10.1016/j.vaccine.2016.08.030 27546875

[82] Kwambana-Adams B Hanson B Worwui A Agbla S Foster-Nyarko E Ceesay F. Rapid Replacement by Non-Vaccine Pneumococcal Serotypes may Mitigate the Impact of the Pneumococcal Conjugate Vaccine on Nasopharyngeal Bacterial Ecology. Sci Rep (2017) 7(1):8127. doi: 10.1038/s41598-017-08717-0 28811633 PMC5557800

[83] Kadioglu A Weiser JN Paton JC Andrew PW. The Role of Streptococcus Pneumoniae Virulence Factors in Host Respiratory Colonization and Disease. Nat Rev Microbiol (2008) 6(4):288–301. doi: 10.1038/nrmicro1871 18340341

[84] Nieto PA Riquelme SA Riedel CA Kalergis AM Bueno SM. Gene Elements That Regulate Streptococcus Pneumoniae Virulence and Immunity Evasion. Curr Gene Ther (2013) 13(1):51–64. doi: 10.2174/156652313804806615 23189947

[85] Rubins JB Pomeroy C. Role of Gamma Interferon in the Pathogenesis of Bacteremic Pneumococcal Pneumonia. Infect Immun (1997) 65(7):2975–7. doi: 10.1128/iai.65.7.2975-2977.1997 PMC175417 9199475

[86] Xia Y Zweier JL. Superoxide and Peroxynitrite Generation From Inducible Nitric Oxide Synthase in Macrophages. Proc Natl Acad Sci USA (1997) 94(13):6954–8. doi: 10.1073/pnas.94.13.6954 PMC21266 9192673

[87] Paczosa MK Mecsas J. Klebsiella Pneumoniae: Going on the Offense With a Strong Defense. Microbiol Mol Biol Rev (2016) 80(3):629–61. doi: 10.1128/MMBR.00078-15 PMC4981674 27307579

[88] Gonzalez-Ferrer S Peñaloza HF Budnick JA Bain WG Nordstrom HR Lee JS. Finding Order in the Chaos: Outstanding Questions in Klebsiella Pneumoniae Pathogenesis. Infect Immun 89(4):e00693–20. doi: 10.1128/IAI.00693-20 PMC8090965 33558323

[89] Farnham A Alleyne L Cimini D Balter S. Legionnaires’ Disease Incidence and Risk Factors, New York, New York, USA, 2002-2011. Emerg Infect Dis (2014) 20(11):1795–802. doi: 10.3201/eid2011.131872 PMC4214295 25513657

[90] Phin N Parry-Ford F Harrison T Stagg HR Zhang N Kumar K. Epidemiology and Clinical Management of Legionnaires’ Disease. Lancet Infect Dis (2014) 14(10):1011–21. doi: 10.1016/S1473-3099(14)70713-3 24970283

[91] Beaute J Zucs P de Jong B European Legionnaires’ Disease Surveillance N. Legionnaires Disease in Europe, 2009-2010. Euro Surveill (2013) 18(10):20417. doi: 10.2807/ese.18.10.20417-en 23515061

[92] Misch EA. Legionella: Virulence Factors and Host Response. Curr Opin Infect Dis (2016) 29(3):280–6. doi: 10.1097/QCO.0000000000000268 26998861

[93] Korf H Vander Beken S Romano M Steffensen KR Stijlemans B Gustafsson JA. Liver X Receptors Contribute to the Protective Immune Response Against Mycobacterium Tuberculosis in Mice. J Clin Invest (2009) 119(6):1626–37. doi: 10.1172/JCI35288 PMC2689129 19436111

[94] Trinh TD Zasowski EJ Claeys KC Lagnf AM Kidambi S Davis SL. Multidrug-Resistant Pseudomonas Aeruginosa Lower Respiratory Tract Infections in the Intensive Care Unit: Prevalence and Risk Factors. Diagn Microbiol Infect Dis (2017) 89(1):61–6. doi: 10.1016/j.diagmicrobio.2017.06.009 28716451

[95] Filloux A. Protein Secretion Systems in Pseudomonas Aeruginosa: An Essay on Diversity, Evolution, and Function. Front Microbiol (2011) 2:155. doi: 10.3389/fmicb.2011.00155 21811488 PMC3140646

[96] Sadikot RT Zeng H Azim AC Joo M Dey SK Breyer RM. Bacterial Clearance of Pseudomonas Aeruginosa is Enhanced by the Inhibition of COX-2. Eur J Immunol (2007) 37(4):1001–9. doi: 10.1002/eji.200636636 17330822

[97] Saliba AM Nascimento DO Silva MC Assis MC Gayer CR Raymond B. Eicosanoid-Mediated Proinflammatory Activity of Pseudomonas Aeruginosa ExoU. Cell Microbiol (2005) 7(12):1811–22. doi: 10.1111/j.1462-5822.2005.00635.x 16309466

[98] Liu XG Li J Zheng LJ Han B Huang F. Interleukin-36 Receptor Antagonist Alleviates Airway Inflammation in Asthma *via* Inhibiting the Activation of Interleukin-36 Pathway. Int Immunopharmacol (2020) 81:106200. doi: 10.1016/j.intimp.2020.106200 32044656

[99] Sun X Hou T Cheung E Iu TN Tam VW Chu IM. Anti-Inflammatory Mechanisms of the Novel Cytokine Interleukin-38 in Allergic Asthma. Cell Mol Immunol (2020) 17(6):631–46. doi: 10.1038/s41423-019-0300-7 PMC7264207 31645649

[100] Tominaga M Okamoto M Kawayama T Matsuoka M Kaieda S Sakazaki Y. Overexpression of IL-38 Protein in Anticancer Drug-Induced Lung Injury and Acute Exacerbation of Idiopathic Pulmonary Fibrosis. Respir Investig (2017) 55(5):293–9. doi: 10.1016/j.resinv.2017.06.001 28942884

[101] Parsanejad R Fields WR Steichen TJ Bombick BR Doolittle DJ. Distinct Regulatory Profiles of Interleukins and Chemokines in Response to Cigarette Smoke Condensate in Normal Human Bronchial Epithelial (NHBE) Cells. J Interferon Cytokine Res (2008) 28(12):703–12. doi: 10.1089/jir.2008.0139 18937544

[102] Kovach MA Che K Brundin B Andersson A Asgeirsdottir H Padra M. IL-36 Cytokines Promote Inflammation in the Lungs of Long-Term Smokers. Am J Respir Cell Mol Biol (2020) 64(2):173–82. doi: 10.1165/rcmb.2020-0035OC PMC7874394 33105081

[103] Chen K Campfield BT Wenzel SE McAleer JP Kreindler JL Kurland G. Antiinflammatory Effects of Bromodomain and Extraterminal Domain Inhibition in Cystic Fibrosis Lung Inflammation. JCI Insight (2016) 1(11). doi: 10.1172/jci.insight.87168 PMC4978187 27517095

[104] Takada K Okamoto T Tominaga M Teraishi K Akamine T Takamori S. Clinical Implications of the Novel Cytokine IL-38 Expressed in Lung Adenocarcinoma: Possible Association With PD-L1 Expression. PloS One (2017) 12(7):e0181598. doi: 10.1371/journal.pone.0181598 28727766 PMC5519175

[105] Scheibe K Kersten C Schmied A Vieth M Primbs T Carle B. Inhibiting Interleukin 36 Receptor Signaling Reduces Fibrosis in Mice With Chronic Intestinal Inflammation. Gastroenterology (2019) 156(4):1082–1097 e11. doi: 10.1053/j.gastro.2018.11.029 30452921

[106] Li W Meng X Hao Y Chen M Jia Y Gao P. Elevated Sputum IL-36 Levels are Associated With Neutrophil-Related Inflammation in COPD Patients. Clin Respir J (2021) 15(6):648–56. doi: 10.1111/crj.13338 33559376

[107] Rafeeq MM Murad HAS. Cystic Fibrosis: Current Therapeutic Targets and Future Approaches. J Transl Med (2017) 15(1):84. doi: 10.1186/s12967-017-1193-9 28449677 PMC5408469

[108] Dittrich AS Kuhbandner I Gehrig S Rickert-Zacharias V Twigg M Wege S. Elastase Activity on Sputum Neutrophils Correlates With Severity of Lung Disease in Cystic Fibrosis. Eur Respir J (2018) 51(3). doi: 10.1183/13993003.01910-2017 29545279

[109] Pan QZ Pan K Zhao JJ Chen JG Li JJ Lv L. Decreased Expression of Interleukin-36alpha Correlates With Poor Prognosis in Hepatocellular Carcinoma. Cancer Immunol Immunother (2013) 62(11):1675–85. doi: 10.1007/s00262-013-1471-1 PMC11029579 24061617

[110] Tsai YC Tsai TF. Anti-Interleukin and Interleukin Therapies for Psoriasis: Current Evidence and Clinical Usefulness. Ther Adv Musculoskelet Dis (2017) 9(11):277–94. doi: 10.1177/1759720X17735756 PMC5764033 29344110

[111] Bachelez H Choon SE Marrakchi S Burden AD Tsai TF Morita A. Inhibition of the Interleukin-36 Pathway for the Treatment of Generalized Pustular Psoriasis. N Engl J Med (2019) 380(10):981–3. doi: 10.1056/NEJMc1811317 30855749

[112] Conner KP Pastuskovas CV Soto M Thomas VA Wagner M Rock DA. Preclinical Characterization of the ADME Properties of a Surrogate Anti-IL-36r Monoclonal Antibody Antagonist in Mouse Serum and Tissues. MAbs (2020) 12(1):1746520. doi: 10.1080/19420862.2020.1746520 32310023 PMC7188401

[113] Leabman MK Meng YG Kelley RF DeForge LE Cowan KJ Iyer S. Effects of Altered FcgammaR Binding on Antibody Pharmacokinetics in Cynomolgus Monkeys. MAbs (2013) 5(6):896–903. doi: 10.4161/mabs.26436 24492343 PMC3896603

[114] Hojen JF Kristensen MLV McKee AS Wade MT Azam T Lunding LP. IL-1r3 Blockade Broadly Attenuates the Functions of Six Members of the IL-1 Family, Revealing Their Contribution to Models of Disease. Nat Immunol (2019) 20(9):1138–49. doi: 10.1038/s41590-019-0467-1 PMC6707854 31427775

[115] Todorovic V Su Z Putman CB Kakavas SJ Salte KM McDonald HA. Small Molecule IL-36gamma Antagonist as a Novel Therapeutic Approach for Plaque Psoriasis. Sci Rep (2019) 9(1):9089. doi: 10.1038/s41598-019-45626-w 31235749 PMC6591177

[116] Camiolo MJ Gauthier M Kaminski N Ray A Wenzel SE. Expression of SARS-CoV-2 Receptor ACE2 and Coincident Host Response Signature Varies by Asthma Inflammatory Phenotype. J Allergy Clin Immunol (2020) 146(2):315–24.e7. doi: 10.1016/j.jaci.2020.05.051 PMC7283064 32531372

[117] Yan R Zhang Y Li Y Xia L Guo Y Zhou Q. Structural Basis for the Recognition of SARS-CoV-2 by Full-Length Human Ace2. Science (2020) 367(6485):1444–8. doi: 10.1126/science.abb2762 PMC7164635 32132184

